# CD47-blocking Antibody ZL-1201 Promotes Tumor-associated Macrophage Phagocytic Activity and Enhances the Efficacy of the Therapeutic Antibodies and Chemotherapy

**DOI:** 10.1158/2767-9764.CRC-22-0266

**Published:** 2022-11-10

**Authors:** Anthony Cao, Jiaqing Yi, Xinyan Tang, Christopher W. Szeto, Renyi Wu, Bing Wan, Xu Fang, Shou Li, Lei Wang, Lina Wang, Jing Li, Qiuping Ye, Tom Huang, Karl Hsu, Omar Kabbarah, Haiying Zhou

**Affiliations:** 1 Zai Lab (US) LLC, Menlo Park, California.

## Abstract

Tumor-associated macrophages (TAM) are the most abundant immune cells in the tumor microenvironment. They consist of various subsets but primarily resemble the M2 macrophage phenotype. TAMs are known to promote tumor progression and are associated with poor clinical outcomes. CD47 on tumor cells and SIRPα on TAMs facilitate a “don't-eat-me” signal which prevents cancer cells from immune clearance. Therefore, blockade of the CD47–SIRPα interaction represents a promising strategy for tumor immunotherapy. Here, we present the results on ZL-1201, a differentiated and potent anti-CD47 antibody with improved hematologic safety profile compared with 5F9 benchmark. ZL-1201 enhanced phagocytosis in combination with standards of care (SoC) therapeutic antibodies in *in vitro* coculture systems using a panel of tumor models and differentiated macrophages, and these combinational effects are Fc dependent while potently enhancing M2 phagocytosis. *In vivo* xenograft studies showed that enhanced antitumor activities were seen in a variety of tumor models treated with ZL-1201 in combination with other therapeutic mAbs, and maximal antitumor activities were achieved in the presence of chemotherapy in addition to the combination of ZL-1201 with other mAbs. Moreover, tumor-infiltrating immune cells and cytokine analysis showed that ZL-1201 and chemotherapies remodel the tumor microenvironment, which increases antitumor immunity, leading to augmented antitumor efficacy when combined with mAbs.

**Significance:**

ZL-1201 is a novel anti-CD47 antibody that has improved hematologic safety profiles and combines with SoC, including mAbs and chemotherapies, to potently facilitate phagocytosis and antitumor efficacy.

## Introduction

Considerable evidence has shown that tumor-associated macrophages (TAM), particularly M2 macrophages, are the most abundant immune cells residing in the tumor microenvironment and are one of the key mechanisms of drug resistance (1, 2). Current strategies to overcome TAM-mediated suppression include inhibiting recruitment, depleting populations, regulating TAM polarization to protumoricidal M1 subtypes, and enhancing tumor-killing abilities, all of which are promising strategies to overcome TAM-related drug resistance (2, 3).

Therapeutic mAbs are the standards of care (SoC) for several hematologic and solid cancers. These include rituximab for CD20-expressing lymphomas, trastuzumab for HER2-amplified cancers, and cetuximab for EGFR-driven solid tumors (4). Although the primary mechanism of action for these therapies is tumor-cell targeting, they are rarely curative, partly because tumor cells employ a variety of mechanisms, including TAMs, to evade immune surveillance and blunt the antitumor immune response (5, 6).

Tumor cells upregulate CD47 expression to facilitate a “don't-eat-me” signal that inhibits macrophage-mediated phagocytosis of tumor cells (4, 5). Currently, agents that disrupt CD47–SIRPα interaction are actively under clinical trials, and promising results have been observed thus far (5–7). However, CD47 is ubiquitously expressed on all cells including erythrocytes and platelets, forming a large antigen sink and leading to potential on-target hematologic toxicities. As a result, off-tumor toxicities such as anemia and thrombocytopenia have been observed in patients subjected to anti-CD47 treatment in clinical studies (8). Therefore, novel CD47-targeting agents with better hematologic safety profiles are needed, and understanding of where and how to use CD47-targeting agents in solid tumors still requires further investigation.

Here, we present preclinical data supporting the combinatorial effects of ZL-1201 and SoC mAbs in hematologic and solid tumor models. ZL-1201 potently blocked CD47–SIRPα interaction and enhanced phagocytosis, as measured by flow cytometry and high-content imaging analysis. Notably, in combination with trastuzumab, cetuximab, or rituximab, ZL-1201 enhanced phagocytosis across hematologic and solid tumor models expressing varying levels of CD47 in *in vitro* coculture systems. Neutralizing the Fc receptor function of these SoC mAbs using mAb-F(ab’)_2_ or blocking CD16, CD32, and CD64 abrogated the combinatorial effects, indicating that CD47 blockade sensitized tumors to prophagocytic signals provided by antibody-dependent cellular phagocytosis (ADCP)-inducing mAbs in an Fc receptor-dependent manner. Furthermore, treatment of xenografts with ZL-1201 in combination with trastuzumab, rituximab, or cetuximab in the absence or presence of chemotherapy drove antitumor immune responses, and remodeled the tumor microenvironment, including increased M1 and decreased M2 macrophages, highlighting the pivotal role of ZL-1201 in overcoming resistance to therapeutic mAbs derived from TAMs.

## Materials and Methods

### Cell Lines

BT474, HCC1954, SKOV3, HCC2218, AU565, Toledo, Raji, Jurkat, FaDu, NCI.H747, SW48, and Cal27 were purchased from ATCC in either 2020 or 2021. ATCC has confirmed the identity of human cell lines by (short tandem repeat analyses. Cells were cultured and maintained in media supplemented with 10% or 20% of FBS per ATCC's recommendation. Fresh stocks of cells were made on early passages and fresh culture of each line was started every 2–3 months for *in vitro* assays. *Mycoplasma* testing was performed on cancer cell lines on July 6, 2022. Primary macrophages were not assessed for *Mycoplasma*.

### Discovery of ZL-1201 Antibody

Four BALB/c and 4 C57BL/6 mice (female, 8 weeks) were immunized with recombinant His-tagged human CD47 protein ranging from Gln19 to Pro139. Animals were given three boosters after the initial immunization. Serum titers were evaluated by ELISA and FACS. Hybridoma was generated by using splenocytes from immunized mice with best *in vitro* binding affinity fused with myeloma cell SP2/0 cells. Variable region genes from positive hybridoma cells were recovered by RT-PCR and then cloned into a standard cloning vector. The variable heavy and light chain (VH/VL) interface was analyzed and mutations were introduced where needed for humanization. The humanized VH/VLs were converted to full length IgG4 format bearing S228P and L235E in the Fc region. All humanized variants were screened in CD47 binding assay, CD47–SIRPα ligand binding, and blocking assay using Raji tumor cell lines.

### Structure of ZL-1201 Fab in Complex with CD47

The coding sequences for His-tagged human CD47 (aa:19–141) and ZL-1201 Fab heavy and light chains were designed and optimized for mammalian cell expression system. Proteins were purified by Ni-EXCEL column or size-exclusion columns. Purified CD47 and ZL-1201 Fab proteins were mixed at 1.2:1 molar ratio at 4°C for 2 hours, then CD47/ZL-1201 complex was purified for crystallization. The diffraction data were collected and processed into highest resolution at 2.2 Å. The structure of CD47 and ZL-1201 Fab complex was solved using molecular replacement method in Phaser and refined using Phenix.Refine.

### Antibodies and Therapeutic Antibodies

Trastuzumab (anti-HER2, human IgG1), cetuximab (anti-EGFR, human IgG1), and rituximab (anti-CD20, IgG1) were acquired from Biointron.

The 5F9 analog was generated according to published sequences (9, 10) and used as a positive control in the *in vitro* and *in vivo* studies and is denoted as a benchmark.

F(ab’)_2_ fragments of trastuzumab, cetuximab, and rituximab were generated by digesting the parental IgG1 antibody with pepsin, according to the manufacturer's instructions [Pierce F(ab’)_2_ Preparation Kit].

### PathHunter CD47–SIRPα Signaling Assay

Evaluation of CD47–SIRPα neutralization was evaluated using a cell-based PathHunter CD47–SIRPα signaling assay (Eurofins). Briefly, 0.75 × 10^6^ CD47-presenting ligand cells were seeded into 96-well microplates and serially diluted anti-CD47 antibody was added to each well, followed by the addition of SIRPα signaling cells. After 24 hours, luciferase activity was measured using a PathHunter Bioassay Detection Kit on a BioTek plate reader.

### CD47–SIRPα Interaction Blockade Assay

FcγR preblocked Raji cells were incubated with antibodies in the presence of EC_80_ concentration of biotinylated SIRPα. Streptavidin-APC conjugate was added for detection after incubation at 4°C for 30 minutes. Mean fluorescence intensity was measured using flow cytometry.

### qRT-PCR

mRNA from the cell lines was extracted using a QIACube Connect (Qiagen). qRT-PCR was performed using SensiFAST SYBR No-ROX One-Step Kit reagents (BIOLINE) on a Bio-Rad CFX384 Real-time qRT-PCR System. The relative mRNA levels of *CD47*, *HER2*, *CD20*, *EGFR*, *CD86*, *CD163*, *CD68*, *CD14*, *CD80*, *CD206*, *CXCL10*, *SIRPA*, and *SIGLEC10* were normalized to the internal control *RPL27*.

### *In Vitro* Binding of ZL-1201 on Tumor Cells

The binding affinity of ZL-1201 for CD47 was determined using flow cytometry. Jurkat CD47-presenting cells were incubated with serially diluted ZL-1201 at room temperature for 15 minutes and washed with staining buffer (BD Pharmingen). After incubation with mouse anti-human IgG4 Fc-FITC (Southern Biotech) for 15 minutes, the cells were washed twice with the stain buffer. Data were collected on a flow cytometer (BD LSR Fortessa X-20) and analyzed using FlowJo software.

### *In Vitro* Binding of ZL-1201 on Human Red Blood Cells and Platelets

Human whole blood was diluted with Dulbecco PBS at a volume ratio of 1:100, serially diluted anti-CD47 mAbs were added to the diluted whole blood and incubated at 4℃ for 30 minutes. Samples were stained with CD61, CD235a, anti-human IgG for 30 minutes at 4℃ in the dark and subjected to flow cytometry. CD235a^+^ cells are indicated as human red blood cells (RBC) and CD61^+^ cells are indicated as platelets. Data are representative of two independent experiments from separate donors and are presented as the mean ± SEM.

### Hemagglutination Assay

A total of 100 μL of serially diluted anti-CD47 mAbs were mixed with 100 μL of 1:50 (volume for volume) prediluted whole blood in a 96-well U-bottom microwell plate and incubated at 37℃/5% CO_2_ for 2 hours for hemagglutination determination.

### Toxicology Study in Cynomolgus Monkeys

Whole blood from 3 cynomolgus monkeys was incubated individually with serial diluted ZL-1201 at various concentrations, binding activity was measured using Alexa Fluor 647 AffiniPure F(ab’)₂ Fragment Goat Anti-Human IgG followed by flow cytometry analysis. Each datapoint represents mean value of duplicate wells. HuIgG4 at 1.33 μM/L is set as a negative control.

Twelve naïve male cynomolgus monkeys were randomly assigned to four groups to determine the toxicity of ZL-1201 or Hu5F9-G4 when administered by intravenous infusion once weekly for 28 days (on days 1, 8, 15, and 22). Monkeys were 2.3–2.7 years of age with body weights ranging from 2.3 to 2.5 kg. The mortality, clinical observations, body weight, food consumption, and clinical pathology (hematology, coagulation, serum chemistry, and urinalysis) were accessed and evaluated.

### Macrophage Differentiation and Polarization

Human peripheral blood mononuclear cells (PBMC) were obtained from STEMCELL Technologies. Monocytes were purified from PBMCs using the EasySep Human Monocyte Isolation Kit (STEMCELL Technologies) and cultured in RPMI containing 10% human AB serum (Thermo Fisher Scientific) and MCSF (10 ng/mL, PeproTech). Fresh medium containing MCSF was added on days 3 and 6. Macrophages were harvested on days 7–10 and denoted as macrophages-derived from monocytes monocyte-derived macrophages (MDM or M0), used immediately for downstream experiments, or cryopreserved in CryoStor10 (STEMCELL Technologies) for later use.

M1 macrophages (M1) were polarized from M0, treated with 100 pg/mL lipopolysaccharide and 50 ng/mL IFNγ for 24 hours and the treatment was withdrawn for 24 hours. M2 macrophages (M2) were polarized from M0 and treated with 10 ng/mL IL10 and 50 ng/mL TGFβ for 48 hours. Polarization was confirmed by flow cytometry; M1 macrophages were CD68^+^CD80^+^CD206^−^ and M2 macrophages were CD68^+^CD163^+^CD206^+^.

### Confocal-based Phagocytosis Assay

BT474, Cal27, and Toledo cells were labeled with carboxyfluorescein diacetate succinimidyl ester (eBioscience) and treated with the following for 15 minutes in 96-well ultralow attachment plate: IgG control (10 μg/mL), ZL-1201 (10 μg/mL), trastuzumab/cetuximab/rituximab (0.1 μg/mL), or their combination. MDMs were added to tumor cells (1:5 E: T) and incubated at 37°C for 3 hours before transferring the cells into the imaging plate (Ibidi) for an additional 2 hours for macrophage attachment, then nonphagocytosed tumor cells were washed away. The attached macrophages were fixed in 4% paraformaldehyde for 15 minutes and then stained with wheat germ agglutinin (Invitrogen). Phagocytosis was assessed by ImageXpress Micro automated microscope (Molecular Devices). The phagocytosis index was calculated as follows: (total number of tumor cells/total number of macrophages) ×100. A minimum of 2,000 macrophages were counted per sample.

### Flow Cytometry Analysis

To measure the cell surface expression of CD47, HER2, EGFR, and CD20, cells were stained with conjugated primary antibodies (BD Biosciences) for 30 minutes at 4°C, washed, and resuspended in staining buffer. Macrophages were incubated with anti-CD16/32 (BD Biosciences) for 15 minutes at room temperature to block nonspecific binding and stained with CD14, CD68, CD80, CD163, CD206, PD-L1, and HLA-DR (BD Biosciences). Cells were acquired using a BD LSR X-20, and the data were analyzed using FlowJo software.

### Multiplex Cytokine/chemokine Immunoassay

Supernatant from polarized M0, M1, or M2 macrophages was collected after 48 hours, and assessed for CXCL10 (PeproTech), MCP1, and IL10 (BioLegend) secretion by sandwich ELISA.

Tumor tissue and serum from xenograft animal models were collected posttreatment at different timepoints for cytokine/chemokine analysis. Tumor tissue was homogenized with Tissue Lyser II (Qiagen) in lysis buffer (Cell Signaling Technology) containing a proteinase inhibitor cocktail (Thermo Fisher Scientific). After ultracentrifugation at 16,000 × *g* for 30 minutes, the soluble matrix was collected from the homogenized mixture. Cytokines and chemokines in the tumor lysates and serum were measured using Luminex or MSD multiplex immunoassays.

### Flow Cytometry–based Phagocytosis Assay

Tumor cells were labeled with CellTrace Oregon Green 488 (Invitrogen) and incubated with antibodies at 37°C in 5% CO_2_ for 15 minutes. Then, human monocyte-derived macrophages (MDM) labeled with CellTrace Far Red (FR; Invitrogen) were added to tumor cells (1:2 E: T) and incubated at 37°C in 5% CO_2_ for 3 hours before analysis. For some experiments, macrophages were incubated with 10 μg/mL anti-CD16 (3G8, BioLegend), anti-CD32 (FLI8.26, STEMCELL), and anti-CD64 (10.1, BioLegend) for 15 minutes to neutralize Fc receptors prior to tumor coculture. Data were collected on a flow cytometer (BD LSR Fortessa X-20) and analyzed using FlowJo software.

### *In Vitro* Phagocytosis of ZL-1201 in Combination with mAbs

CellTrace Oregon Green (OG)-labeled human cancer cells, including HCC1954, SKOV3, BT474, HCC2218, AU565, NCI.H747, SW48, FaDu, Cal27, Raji, or Toledo were plated (6 × 10^4^) in a 96-well plate and treated with isotype control (10 μg/mL), ZL-1201 or benchmark anti-CD47 (10 μg/mL), and trastuzumab/cetuximab/rituximab (0.1 μg/mL) separately or in combination as indicated and incubated for 30 minutes. CellTrace FR-labeled human MDMs were added (3 × 10^4^) to tumor cells and cocultured for 3 hours before flow cytometry analysis. The percent of MDM phagocytosis+ was calculated from the ratio of OG+/FR+ to total FR+ macrophages. Fold phagocytosis in the treated groups was calculated relative to the isotype control.

### *In Vivo* Xenograft Studies

Each mouse was inoculated subcutaneously at the right flank with Raji tumor cells (3 × 10^6^/mouse). A total of 10 mg/kg, i.p., twice per week*3 weeks. Hu5F9 was selected as a reference antibody brought from Biointron (catalog no. B3048, lot no. 20181013A02), and human IgG4 was set as negative control. The tumor volumes were measured three times per week using standard caliper. Datapoints represent group mean; error bars represent SEM.

Raji, HCC1954, SKOV3, and FaDu cells were maintained *in vitro* as described previously and inoculated subcutaneously into BALB/c nude mice in the upper flank. When tumors reached 100 mm^3^, the mice were randomized and treatment was initiated. The Raji cohort was inoculated with 1 × 10^7^ cells and treated with 10 mg/kg of the isotype or ZL-1201 intraperitoneally twice per week, 10 mg/kg of rituximab intraperitoneally once per week, or their combination for 3 weeks.

The HCC1954 cohort was inoculated with 5 × 10^6^ cells in PBS mixed with Matrigel and treated with 10 mg/kg of isotype control or ZL-1201 intraperitoneally, twice per week, 1 mg/kg of herceptin intraperitoneally, once per week, 2 mg/kg of docetaxel, intraperitoneally, once per week, or their combinations for approximately 8–12 weeks.

The SKOV3 cohort was inoculated with 1 × 10^7^ cells in PBS mixed with Matrigel and treated with 10 mg/kg of the isotype control or ZL-1201 intraperitoneally twice per week, 2 mg/kg of herceptin intraperitoneally, once per week, 7.5 mg/kg of paclitaxel, intravenously, once per week, or their combinations for approximately 5–7 weeks.

The FaDu cohort was inoculated 5 × 10^6^ and treated with 10 mg/kg of the isotype control or ZL-1201, intraperitoneally, twice per week, 0.5 mg/kg of erbitux intraperitoneally, once per week, 5 mg/kg of cisplatin, intravenously, once per week, or their combinations for approximately 4–9 weeks.

The patient-derived xenograft (PDX) model of ST-02-0077 was originally established from a surgically resected clinical sample and was implanted and passaged in nude mice. BALB/c nude mice were implanted subcutaneously in the right flank with PDX tumor slices (passage 4, ∼30 mm^3^). When the average tumor size reached 150–200 mm^3^, mice were randomized and treated with 10 mg/kg of the isotype or ZL-1201 intraperitoneally twice per week, 5 mg/kg of herceptin, intraperitoneally, or twice per week. A total of 4 mg/kg of docetaxel, intravenously, twice per week, or their combinations, as shown for approximately 6 weeks.

Tumor sizes were measured twice per week and tumor growth curves are shown as mean + SEM. Student *t* test was used for statistical analysis to compare the tumor volumes of distinct groups on a specific day. All tests were two sided unless otherwise specified, and *P* values <0.05 were regarded as statistically significant.

### Flow Cytometry Analysis on Tumor Tissues

At the end of the SKOV3 efficacy study, tumors from each treatment group were collected 96 hours after the last treatment. Single-cell suspensions were generated using a GentleMACS Octo Dissociator and Tumor Dissociation Kit (Miltenyi Biotec). The cells were strained through a 70 μm cell strainer and incubated with anti-CD16/32 (BD Fc Block) for 10 minutes at 4°C. The cells were then stained for CD45, CD11b, CD103, Ly6G, CD80 (BD Biosciences), CD335, F4/80, I^A^-I^E^ (MHCII), CD206, CD11c, Ly6c, PD-L1, SIRPα (BioLegend), and viability dye (eBioscience) for 30 minutes at 4°C. Samples were collected on an the LSR Fortessa X-20 (BD) and analyzed using Flowjo software.

### Animal Welfare and Regulatory Compliance

The protocol and any amendment(s) or procedures involving the care and use of animals in this study were reviewed and approved by the Institutional Animal Care and Use Committee (IACUC) of Crown Bioscience and WuXi AppTec (HongKong) Limited prior to execution. All studies were conducted in accordance with the approved IACUC protocol. During the study, animal care and use were conducted following the regulations of the Association for Assessment and Accreditation of Laboratory Animal Care.

### Genomic Immune Profiling Analysis

To identify tissue types with sufficient macrophage presence for CD47-targeted agents, we obtained CIBERSORTscores in arbitrary units that reflect the absolute proportion of each cell type, inferred immune infiltrate scores from The Cancer Genome Atlas (TCGA) sources (11) for >11 K patients from across 31 tissue types. The total macrophage fraction was estimated for each patient by adding the M0, M1, and M2 macrophage scores. Among the 31 tissue types, 13 were defined as “high total macrophage” types based on the majority of patients with total macrophages higher than the median across TCGA. To show that the 13 tissue types selected for high macrophage infiltration had elevated M2 macrophages specifically, the ratio of the M2:M1 macrophages was compared between high- and low-macrophage tissue types. To avoid division by zero, the M2:M1 ratio was defined as log(M2+1) − log(M1+1), where M2 and M1 are CIBERSORT-ABS scores for macrophage (M2) and macrophage (M1), respectively. To assess the prognostic value of M2:M1 ratio, the median M2:M1 ratio within each indication was used to subgroup the patients into low- and high-M2:M1-ratio groups. Updated progression-free interval (PFI) data were obtained from Liu and colleagues (12) and used to test differential survival in the M2:M1-ratio group log-rank test.

There are 48 genes in the gene signature for macrophage (M1) and 37 genes for macrophage (M2). These genes are list below and can also be found from the CIBORSORT publication (13).

Macrophage (M1) gene signature: *ACHE*, *ADAMDEC1*, *APOBEC3A*, *APOL3*, *APOL6*, *AQP9*, *ARRB1*, *CCL19*, *CCL5*, *CCL8*, *CCR7*, *CD38*, *CD40*, *CHI3L1*, *CLIC2*, *CXCL10*, *CXCL11*, *CXCL13*, *CXCL9*, *CYP27B1*, *DHX58*, *EBI3*, *GGT5*, *HESX1*, *IDO1*, *IFI44L*, *IL2RA*, *KIAA0754*, *KYNU*, *LAG3*, *LAMP3*, *LILRA3*, *LILRB2*, *NOD2*, *PLA1A*, *PTGIR*, *RASSF4*, *RSAD2*, *SIGLEC1*, *SLAMF1*, *SLC15A3*, *SLC2A6*, *SOCS1*, *TLR7*, *TLR8*, *TNFAIP6*, *TNIP3*, *TRPM4*

Macrophage (M2) gene signature: *ADAMDEC1*, *AIF1*, *ALOX15*, *CCL13*, *CCL14*, *CCL18*, *CCL23*, *CCL8*, *CD209*, *CD4*, *CD68*, *CFP*, *CHI3L1*, *CLEC10A*, *CLEC4A*, *CLIC2*, *CRYBB1*, *EBI3*, *FAM198B*, *FES*, *FRMD4A*, *FZD2*, *GGT5*, *GSTT1*, *HRH1*, *HTR2B*, *MS4A6A*, *NME8*, *NPL*, *P2RY13*, *PDCD1LG2*, *RENBP*, *SIGLEC1*, *SLC15A3*, *TLR8*, *TREM2*, *WNT5B*

### Data Availability Statement

TCGA data supporting the immunogenomic profiling are available at: https://api.gdc.cancer.gov/data/b3df502e-3594–46ef-9f94-d041a20a0b9a.

## Results

### ZL-1201 is a Differentiated Anti-CD47 Therapeutic Antibody with Improved Hematologic Safety Profile

**FIGURE 1:**
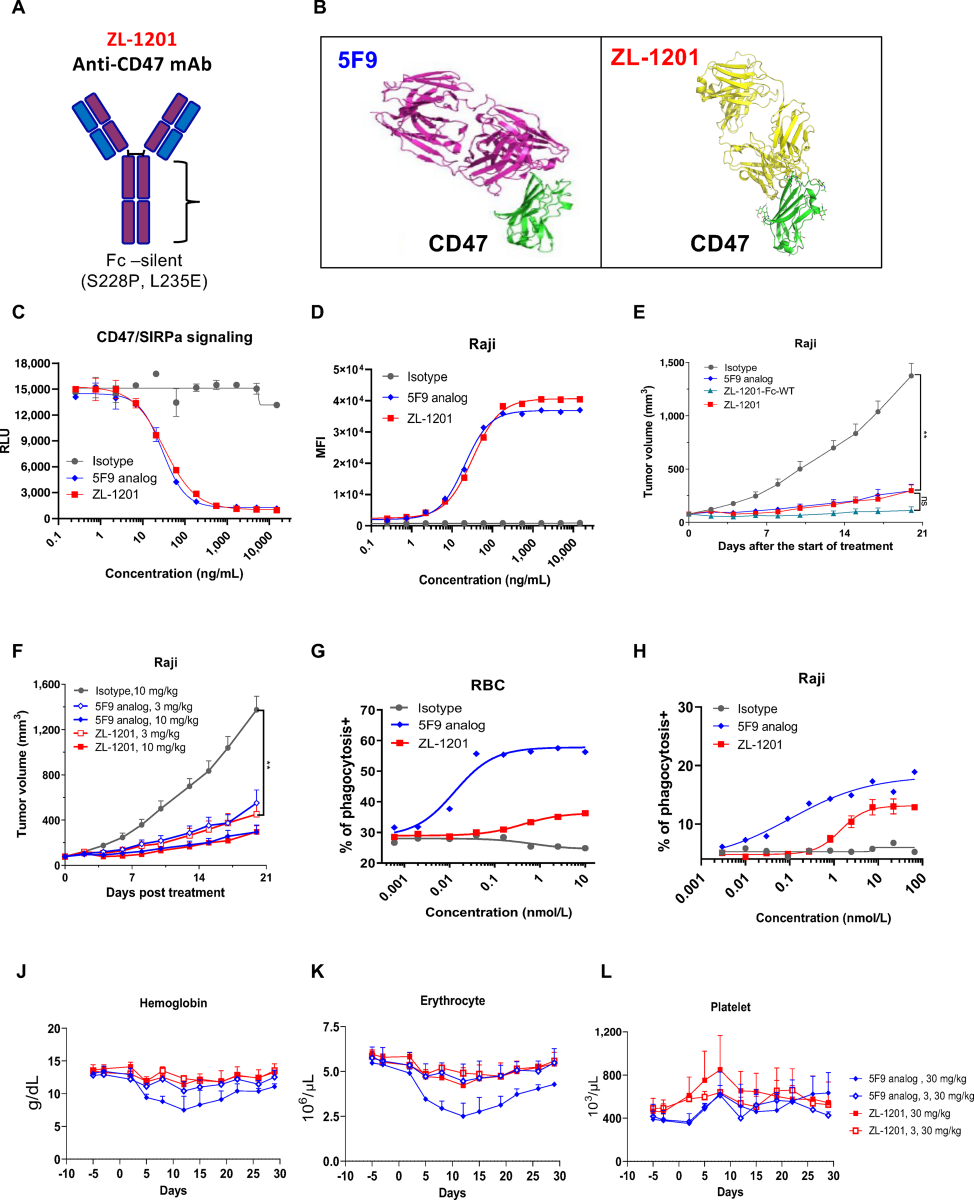
ZL-1201 is a differentiated anti-CD47 therapeutic antibody with improved hematologic safety profile. **A,** Scheme of ZL-1201 anti-CD47 mAb. **B,** Structure of 5F9 and ZL-1201 in complex with CD47. **C,** ZL-1201 comparably disrupts CD47–SIRPα signaling to the benchmark 5F9 analog in a dose-dependent manner. **D,***In vitro* binding of isotype control, benchmark, and ZL-1201 with Raji cells in a dose-dependent manner. **E,** 5F9, ZL-1201-Fc-WT, and ZL-1201 comparative antitumor efficacy study in Raji tumor cell xenograft model. **F,** ZL-1201 and 5F9 comparative dose-dependent antitumor efficacy in Raji tumor cell xenograft model. **G,***In vitro* phagocytosis of RBCs by MDMs with isotype control, benchmark, and ZL-1201 in a dose-dependent manner. **H,***In vitro* phagocytosis of Raji cells by MDMs with benchmark, and ZL-1201 in a dose-dependent manner. The levels of hemoglobin (**J**), erythrocyte counts (**K**), and platelet counts (**L**) were measured in 28-day toxicology studies in cynomolgus monkeys treated with 30 mg/kg of ZL-1201 or benchmark IV (*n* = 3), once per week in the presence or absence of the 3 mg/kg priming dose. Data are presented as mean + SEM.

ZL-1201 was discovered by immunizing and boosting mice with recombinant human CD47 protein, and hybridomas were generated using splenocytes from mice with the best *in vitro* binding affinity (Fig. 1; Supplementary Fig. S1). Selected mouse clones were further humanized into full-length IgG4 format with two mutations (S228P and L235E) in the Fc region to further diminish the weak effector functions of IgG4 antibodies (14), including antibody-dependent cellular cytotoxicity, ADCP, and complement-dependent cytotoxicity (Fig. 1A and B). Compared with Fc wild-type (ZL-1201-Fc-WT), ZL-1201 has similar binding affinity to CD47 (Supplementary Fig. S1E) and similar antitumor response in Raji xenografts (Fig. 1E). As a result, ZL-1201 was developed as a recombinant humanized monoclonal IgG4 antibody that specifically targets CD47 (Fig. 1A). 

Hu5F9 is the most advanced anti-CD47 in clinical development, therefore 5F9 analog was acquired on the basis of the released Hu5F9-G4 sequence (9, 10) and was used as the benchmark in this study. Crystal structures showed that ZL-1201 binds a partial overlapping but distinct epitope on human CD47 (Fig. 1B). The alignment of the complex of ZL-1201/CD47 and that of 5F9/CD45 (PDB5IWL) revealed that ZL-1201 is tilted approximately 15° toward the central axis in XY-plane and rotated backward in Z-plane (Supplementary Fig. S1A). We further compared ZL-1201 with the benchmark in a variety of preclinical *in vitro* studies, and the data showed that ZL-1201 binds CD47 on multiple cancer cells, RBCs, and platelets (Fig. 1D; Supplementary Fig. S1C and S1D; Supplementary Fig. S1F and S1G), and blocks the interaction between CD47 and SIRPα (Fig. 1C; Supplementary Fig. S1B) with potency similar to that of the benchmark. Because of its mutated Fc region, ZL-1201 displayed modest macrophage-mediated phagocytosis of cancer cells compared with the benchmark (Fig. 1H). More importantly, ZL-1201 induced minimal RBC phagocytosis (Fig. 1G) and did not induce hemagglutination in whole blood (Supplementary Fig. S1H). Furthermore, *in vivo* studies demonstrated that ZL-1201 has potent and comparable antitumor efficacy in Raji xenografts to benchmark (Fig. 1E and F).

To evaluate whether ZL-1201 achieved improved hematologic safety profile in comparison with the benchmark, 12 naïve male cynomolgus monkeys were randomly assigned to four groups (*n* = 3) to determine the toxicity of ZL-1201 or benchmark when administered by intravenous infusion once weekly for 28 days. The clinical observations including mortality, body weight, food consumption, and clinical pathology (hematology, coagulation, serum chemistry, and urinalysis) were measured and evaluated. The data showed that ZL-1201 improved hematologic safety profile compared with the benchmark (Fig. 1J–L; Supplementary Fig. S1I–S1K).

In conclusion, ZL-1201 represents a differentiated and effective CD47-targeting therapy with an improved hematologic safety profile.

### ZL-1201 Enhanced Phagocytosis in Combination with mAbs *In Vitro*

**FIGURE 2:**
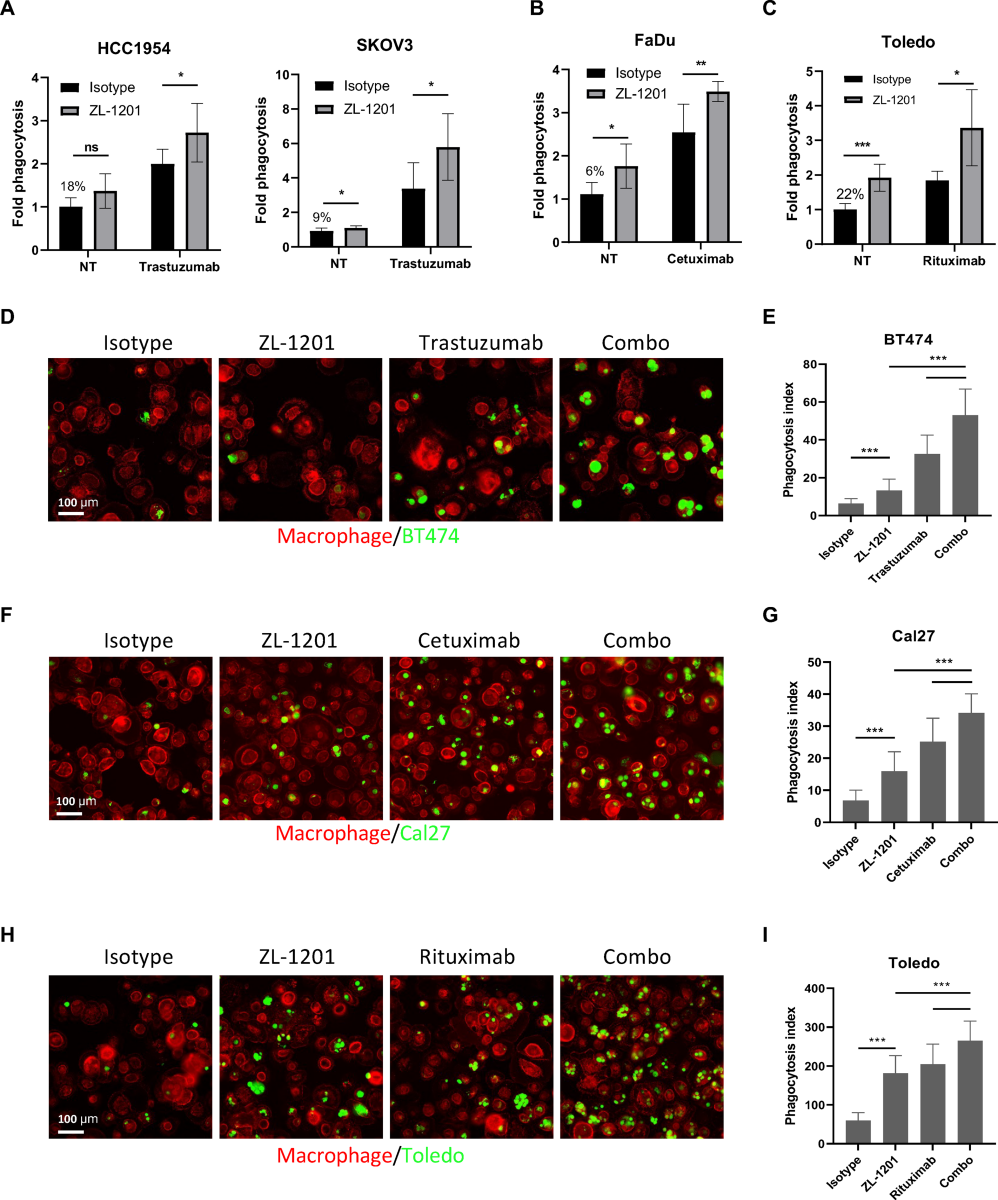
ZL-1201 synergizes with mAbs for potent tumor cell elimination. **A,***In vitro* phagocytosis of HER2-amp solid tumor cells including HCC1954 and SKOV3 treated with isotype control (10 μg/mL), ZL-1201 (10 μg/mL), trastuzumab (0.1 μg/mL), or their combination. **B,***In vitro* phagocytosis of EGFR-driven FaDu HNSCC cells treated with isotype control (10 μg/mL), ZL-1201 (10 μg/mL), cetuximab (0.1 μg/mL), or their combination. **C,***In vitro* phagocytosis of CD20-expressing Toledo lymphoma cells treated with isotype control (10 μg/mL), ZL-1201 (10 μg/mL), rituximab (0.1 μg/mL), or their combination as indicated. Fold phagocytosis in **A** and **B** was calculated relative to isotype in nontreated (NT) conditions, the raw phagocytosis percentages are indicated in each figure. Data were shown as mean ± SD. *, *P* < 0.05; **, *P* < 0.01; ***, *P* < 0.001. *In vitro* phagocytosis was performed in *in vitro* coculture system using macrophage (red), BT474 (**D**), Cal27 (**F**), and Toledo (**H**) (green) cancer cells and photographed by confocal imaging. **E, G,** and **I,** Phagocytosis index was calculated from **D, F,** and **H**. Data were shown as mean ± SD. *, *P* < 0.05; **, *P* < 0.01; ***, *P* < 0.001.

Earlier studies have shown that the antitumor effects of mAbs are augmented by CD47-targeting agents through ADCP (15, 16). To explore whether the combination effects exist between ZL-1201 and mAbs, including trastuzumab, cetuximab, and rituximab (Fig. 2; Supplementary Fig. S2 and S3), we identified cell lines from indications treated with these mAbs as SoCs in the clinic (Supplementary Fig. S2A–S2E). Using flow cytometry and high-throughput confocal imaging approaches, we tested ZL-1201 in combination with trastuzumab in HER2-amplified solid tumor models including HCC1954, SKOV3, BT-474, HCC2218, AU565 (Fig. 2A; Supplementary Fig. S2F), cetuximab in EGFR-expressing head and neck squamous cell carcinoma (HNSCC) and colorectal cancer models including FaDu, Cal27, NCI.H747, and SW48 (Fig. 2B; Supplementary Fig. S2G; Supplementary Fig. S3F), and rituximab in the CD20-expressing Toledo lymphoma cell (Fig. 2C; Supplementary Fig. S3G). The results showed that ZL-1201 improved phagocytosis by flow cytometry (Fig. 2A–C; Supplementary Fig. S2F–S2G) and immunofluorescence (Fig. 2D–I) when combined with therapeutic mAbs. Similar combination effects were observed on benchmark (Supplementary Fig. S3A–S3C). As mAbs drive strong ADCP effects in *in vitro* phagocytosis assays, we used suboptimal doses of mAbs in combination with a high dose of ZL-1201 to visualize the combination effects. In Toledo and FaDu cells, ZL-1201 showed a potent combination with rituximab or cetuximab across a wide dose range (Supplementary Fig. S3D, S3E, and S3H). Taken together, these results suggest that anti-CD47 antibodies enhance phagocytosis in combination with mAbs in a variety of cancer models.

### The Combinational Effects of ZL-1201 with mAbs are Fc dependent

**FIGURE 3:**
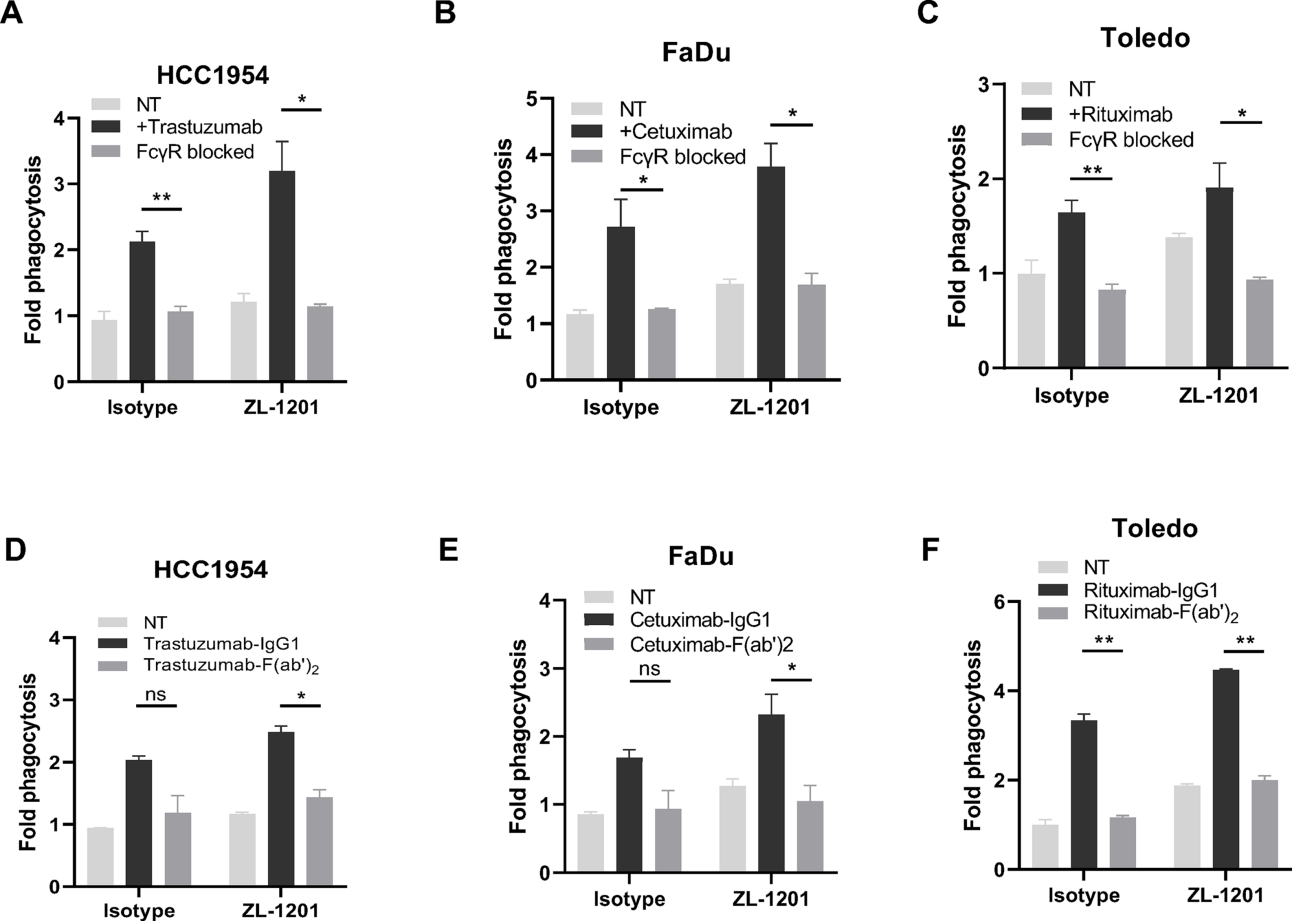
ZL-1201 combination relies on the intact Fc domain on mAbs. Blockade of Fcγ receptors on macrophages limited *in vitro* phagocytosis of HCC1954 (**A**), FaDu (**B**), or Toledo (**C**) treated with isotype control (NT) or ZL-1201 (10 μg/mL) in combination with trastuzumab (**A**), cetuximab (**B**) or rituximab (**C**; 0.1 μg/mL). Treatment with F(ab’)_2_ of trastuzumab (**D**), cetuximab (**E**), or rituximab (**F**) does not enhance phagocytosis of HCC1954 (**D**), FaDu (**E**), or Toledo (**F**) cells in combination with ZL-1201. Data was shown as mean ± SD. *, *P* < 0.05; **, *P* < 0.01.

The Fc region of human IgG1 antibodies supplies a prophagocytic “eat-me” signal to mediate antitumor immunity (6, 17, 18). We hypothesized that coupling the prophagocytic Fc domain with the inhibition of the CD47 checkpoint is critical for maximal phagocytosis. To test whether the combinatorial effects of ZL-1201 and mAbs are Fc dependent, we performed *in vitro* phagocytosis while inhibiting the functions of mAb Fc. Neutralizing the FcγRs on macrophages using anti-CD16, anti-CD32, and anti-CD64 antibodies (Fc blockade) ablated the combinatorial effects of mAb with ZL-1201. In addition, treatment with F(ab’)_2_ fragments did not enhance ZL-1201–mediated phagocytosis, confirming that the intact Fc region of mAbs is essential for maximal phagocytosis by ZL-1201 (Fig. 3; Supplementary Fig. S4).

### ZL-1201 Promoted Phagocytosis by Macrophages Particularly M2 Macrophage in Combination with mAbs

**FIGURE 4:**
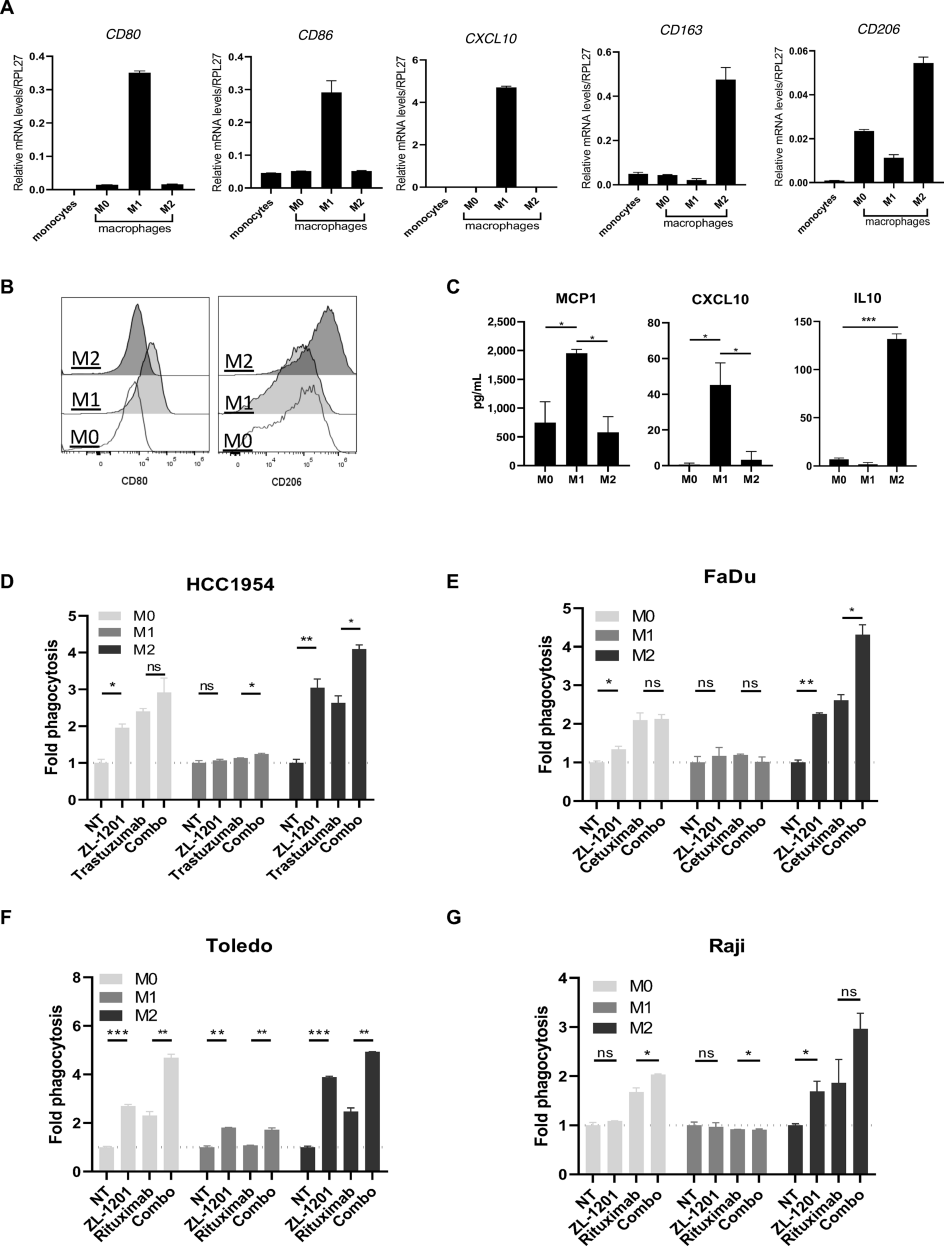
M2 macrophages are highly responsive to ZL-1201 and CD47 blockade. **A,***CD80*, *CD86*, *CXCL10*, *CD163*, and *CD206* transcript levels in monocytes and differentiated M0, M1, and M2 macrophages. **B,** Flow cytometric histogram photos showing CD80 and CD206 surface protein levels in differentiated M0, M1, and M2 macrophages. **C,** MCP1, CXCL10, and IL10 secretion by differentiated M0, M1, and M2 macrophages. *In vitro* phagocytosis using HCC1954 (**D**), FaDu (**E**), Toledo (**F**), and Raji (**G**) cancer cells and differentiated M0, M1, or M2 macrophages under the treatment conditions as shown. Fold phagocytosis was calculated relative to nontreated (NT) group and shown as mean ± SD. *, *P* < 0.05; **, *P* < 0.01; ***, *P* < 0.001.

TCGA immunogenomic analysis showed that M2 macrophages are dominant in most solid tumors and a higher M2:M1 ratio was found to be a significant poor prognostic indicator of PFI in colorectal cancer, breast cancer, and gastric cancers (Supplementary Fig. S5). Previous studies indicated that M2 macrophages are a highly immunosuppressive and protumorigenic subtype of macrophages (19). Intense efforts have focused on manipulating TAMs to modulate the tumor microenvironment or increase antitumor activity. Therefore, we sought to explore whether ZL-1201 specifically enhances the phagocytic abilities of M2 macrophages. To this end, macrophages (M0) were first differentiated from isolated monocytes and further polarized into M1 and M2 subtypes. M1/M2 polarization through expression of *CD80*, *CD86*, *CXCL10*, *CD163*, *CD68*, *CD14*, *CD80*, and *CD206* was assessed by qRT-PCR or flow cytometry analysis (20), as well as production of CXCL10, MCP1, and IL10. These results confirmed the identity and specialized immune functions of each subtype (Fig. 4A–C; Supplementary Fig. S6A and S6B). Next, *in vitro* phagocytosis was performed to compare phagocytosis between macrophage subtypes (M0, M1, and M2) in different tumor cell lines during treatment with ZL-1201 and/or mAbs. The results showed that M1 were highly phagocytic compared with M0 and M2 macrophages. However, the addition of ZL-1201 greatly promoted the phagocytic ability of M2 macrophages, to a level comparable with that of M1 macrophages (Fig. 4D–G; Supplementary Fig. S6C–S6F), indicating that ZL-1201 can restore phagocytic function to multiple subtypes of macrophages, and this effect was not due to the different surface SIRPα levels or M2 phenotype (Supplementary Fig. S6B; Supplementary Fig. S6G). Importantly, ZL-1201 alone or in combination with mAbs strongly promoted phagocytosis by M0 and M2 macrophages (Fig. 4D–G) indicating that ZL-1201 and CD47 inhibition can potently transform poorly phagocytic macrophages into highly phagocytic cells, with maximal killing in combination with mAbs. Notably, ZL-1201 and mAbs had limited effects on phagocytosis by M1 cells, likely due to their potent intrinsic phagocytic activity at baseline.

### ZL-1201 Enhanced Antitumor Activities of Other Therapeutic mAbs in Multiple Xenograft Models with or Without Chemotherapy

**FIGURE 5:**
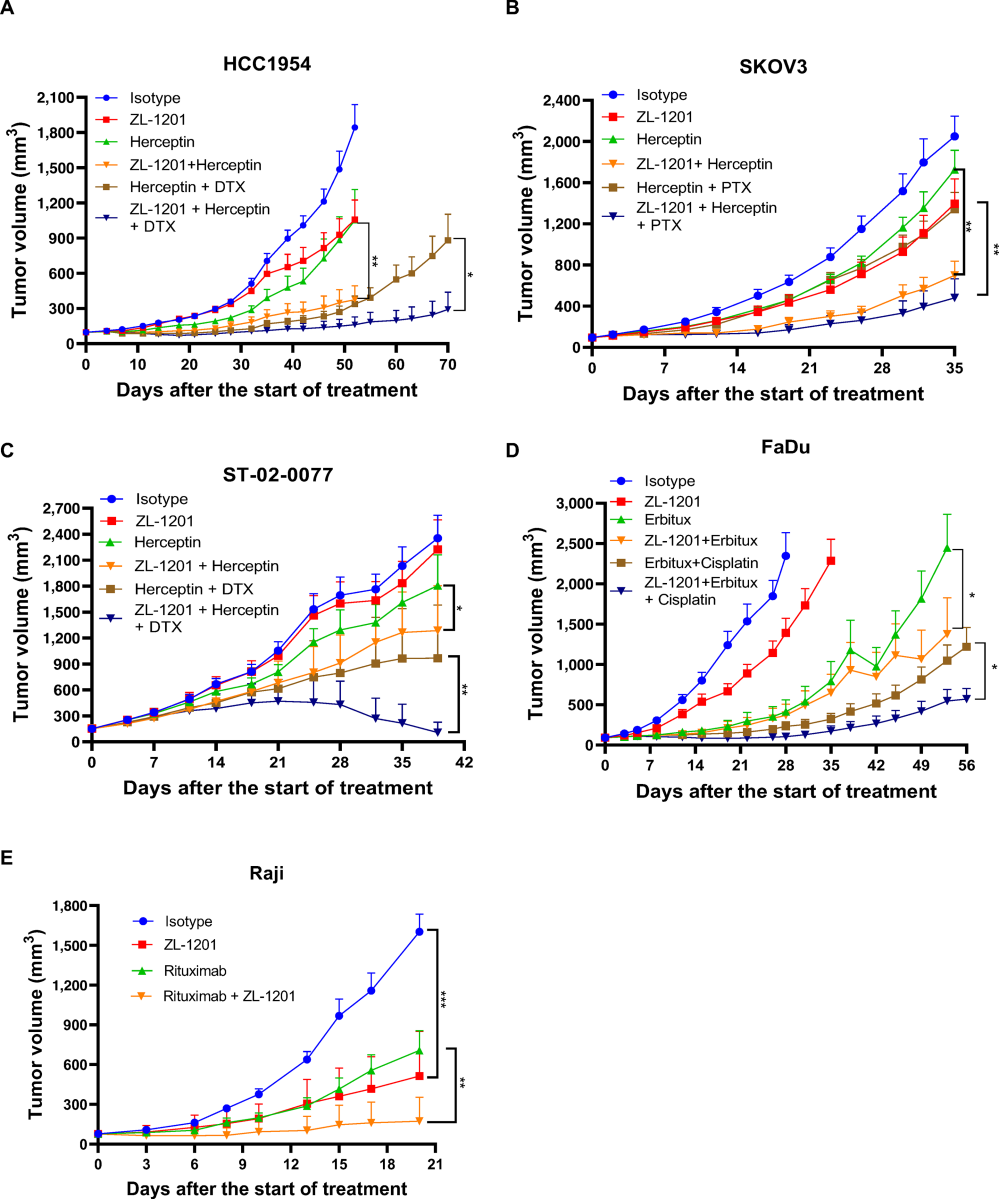
ZL-1201 combines with SoC therapeutics and increased efficacy *in vivo*. Antitumor activities of isotype control, ZL-1201, herceptin, docetaxel (DTX; **A** and **C**) or paclitaxel (PTX; **B**), or their combinations as indicated on HCC1954 (**A**), SKOV3 (**B**) breast cancer xenograft models, and on ST-02-0077 gastric PDX model (**C**). **D,** Antitumor activities of isotype control, ZL-1201, erbitux, cisplatin, or their combinations as shown in FaDu HNSCC xenograft model. **E,** Antitumor activities of isotype control, ZL-1201, rituximab, or their combinations as indicated in Raji lymphoma cancer xenograft model. Tumor sizes were measured twice per week and tumor growth curves were shown as mean + SEM. *, *P* < 0.05; **, *P* < 0.01.

Next, we tested ZL-1201 in combination with mAbs in the presence or absence of chemotherapy in xenograft models in nude mice with functional macrophages. ZL-1201 was combined with herceptin and taxanes to evaluate HCC1954 (breast), SKOV3 (ovarian), and ST-02-0077 (gastric; Fig. 5A–C) models; erbitux and cisplatin to evaluate the FaDu (HNSCC) model (Fig. 5D), and rituximab to evaluate the Raji lymphoma model (Fig. 5E). To explore the combinatorial effects of ZL-1201, suboptimal doses of herceptin, erbitux, and chemotherapeutics were used to limit tumor growth inhibition (TGI) to under 50% in the solid tumor models and provide a therapeutic window for ZL-1201 combination (Fig. 5A–E). Tumor growth curves and individual tumor volumes showed that ZL-1201 significantly inhibited tumor growth and prolonged treatment response in combination with mAbs in the absence or presence of chemotherapy (Fig. 5A–E; Supplementary Fig. S7). Notably, there were no significant body weight changes among the treatment groups in all these studies (Supplementary Fig. S8). Together, these results suggest that ZL-1201 enhanced the antitumor activities of mAbs with or without chemotherapy, while the triple combination achieved maximal antitumor efficacy in all solid tumor xenografts (Fig. 5A–D).

### ZL-1201 Modulated the Tumor Microenvironment Favors Antitumor Activities *In Vivo*

**FIGURE 6:**
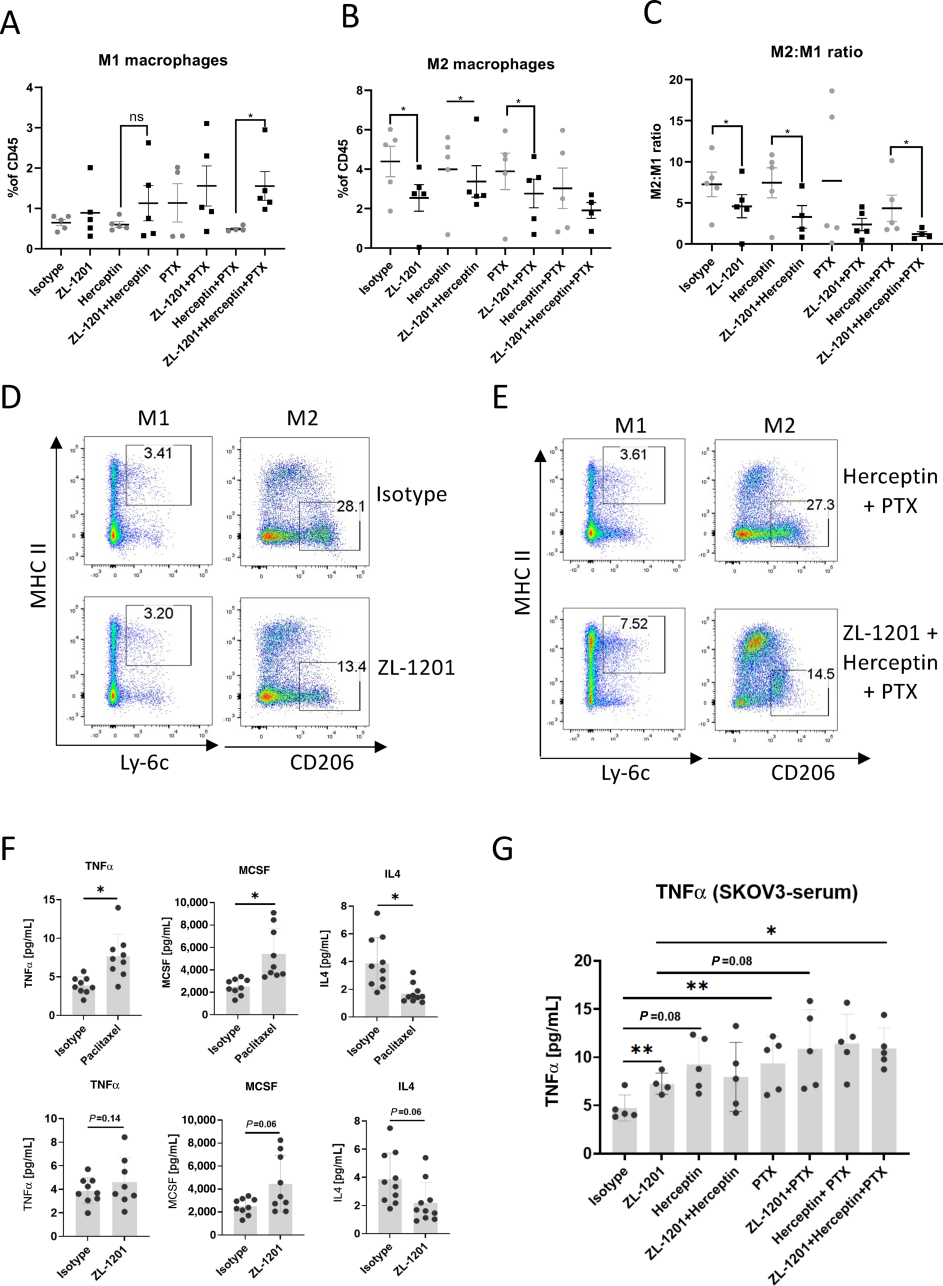
ZL-1201 modulates intratumoral macrophages. Flow cytometry analysis showing percentage of M1 macrophages (**A**), M2 macrophages (**B**), and M2:M1 ratios (**C**) within infiltrating CD45^+^ immune cells in the indicated treatment groups in tumor samples collected at the end of efficacy study of SKOV3 xenograft model as shown in Fig. 5B. **D** and **E**, Flow plots showing M1 and M2 macrophages in one of five SKOV3 xenograft tumor tissues treated as shown. **F,** TNFα, MCSF, and IL4 production were measured by Luminex on SKOV3 tumor tissues 96 hours after paclitaxel or ZL-1201 treatment. **G,** TNFα was quantified in SKOV3 serum at 24 hours after isotype, ZL-1201, herceptin, or paclitaxel treatment. Data are shown as mean ± SD. *, *P* < 0.05; **, *P* < 0.01; ***, *P* < 0.001.

To understand the mechanisms of action of the combined therapeutics, tumors were analyzed for immune-related phenotypic changes at the end of the efficacy study using the SKOV3 model (Fig. 6). The results showed that ZL-1201 treatment increased the frequency of M1 macrophages and decreased M2 macrophages, skewing the M2/M1 ratio toward a more favorable profile (Fig. 6A–C; Supplementary Fig. S9A–S9C). These results suggest a role for ZL-1201 in modulating the TME to increase infiltration by prophagocytic M1-like macrophages. The triple combination group showed profound changes in the macrophage populations, which were concordant with maximal antitumor efficacy. Furthermore, ELISA analysis of tumors and sera showed that ZL-1201, mAbs, and chemotherapy or their combinations elicited the release of proinflammatory cytokines TNFα and MCSF and decreased immunosuppressive IL4 release (Fig. 6E and F), reflecting an increase in M1 macrophages and antitumor immunity. Consistent with *in vitro* modeling, infiltrating macrophages did not demonstrate any difference in SIRPα expression between M1 or M2 macrophages, nor was expression modified by ZL-1201 administration (Supplementary Fig. S9D–S9F).

## Discussion

Through immunogenomic profiling of TCGA, we identified that M2 macrophages are highly enriched among TAMs/macrophages in a variety of solid tumors and that a high M2:M1 ratio predicts worse clinical outcomes in breast, gastric, and colorectal cancers. Therefore, disruption of the CD47–SIRPα interaction is a promising approach for overcoming TAM-derived suppression in a variety of solid tumors.

**FIGURE 7:**
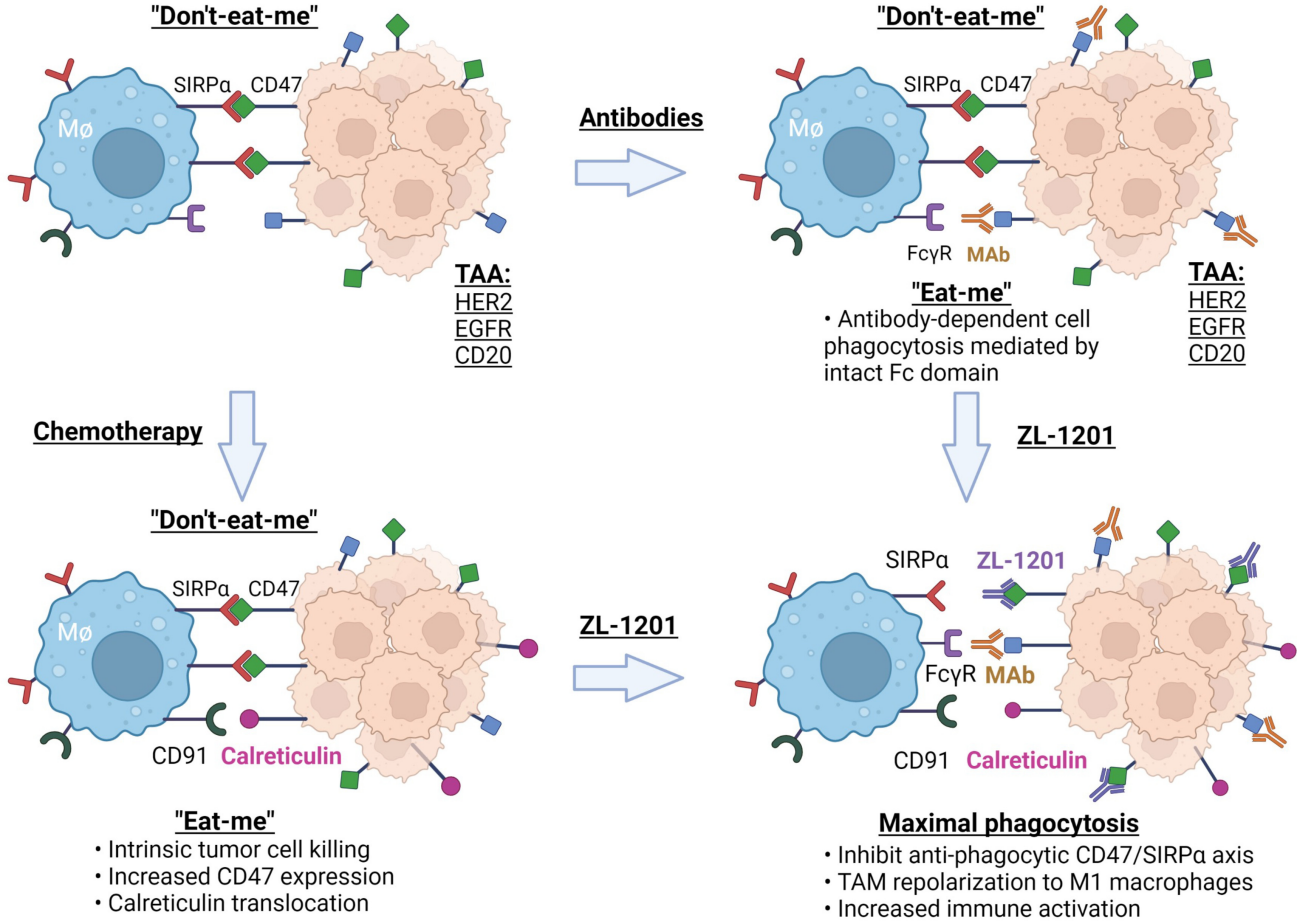
A model showing the mechanisms of action of antitumor combination effects from ZL-1201 in combination with therapeutic mAbs in the presence or absence of chemotherapy. (Created with BioRender.com)

ZL-1201 has potency similar to that of 5F9 in binding CD47 to disrupt the CD47–SIRPα interaction. However, the silenced Fc region decreased the interaction between ZL-1201 and FcγR on effector cells, limiting responses by macrophages, natural killer (NK) cells, and dendritic cells, while improving hematologic safety profiles. Given that ZL-1201 monotherapy showed limited phagocytosis of cancer cell lines *in vitro*, combination therapy is needed to achieve optimal antitumor efficacy when an Fc-silenced IgG4 antibody is applied. Our data showed that ZL-1201 in combination with SoC promoted phagocytosis *in vitro* and delayed tumor growth *in vivo*. Mechanistic studies on tumor-infiltrating immune cells and cytokines/chemokines showed that ZL-1201 neutralizes CD47–SIRPα antiphagocytic signaling, and combination with Fc-intact mAbs further provide a prophagocytic signal to maximally induce potent ADCP and promote tumor clearance (Fig. 7). Chemotherapy and ZL-1201 treatment elicited a panel of proinflammatory cytokines and chemokine release; meanwhile, they inhibited the release of anti-inflammatory cytokines such as IL4, resulting in increased antitumor immunity and macrophage polarization to the M1 subtype (Fig. 6).

Targeting SIRPα is an orthogonal approach to disrupt the CD47–SIRPα interaction and facilitate phagocytosis. SIRPα is mainly expressed in myeloid cells and neurons, anti-SIRPα has a limited antigen sink effect compared with anti-CD47 due to lack of expression on blood cells. Therefore, anti-SIRPα is not expected to cause hematologic toxicities such as anemia, thrombocytopenia, and hemagglutination. However, due to its expression in neurons, potential neuronal toxicities could be a new liability of targeting SIRPα. In addition, the expression of SIRPα homologs, including SIRPβ and SIRPγ might function redundantly in place of SIRPα (21) leading to suboptimal inhibition of the CD47 checkpoint. As a strategy, targeting SIRPα needs further investigation and validation in the clinical settings (22).

Our *in vitro* studies revealed that ZL-1201 substantially enhanced phagocytosis by M2 macrophages, but not by M1 macrophages. The high phagocytic capabilities of M1 cells at baseline may account for this observation. SIRPα is expressed similarly in M1 and M2 macrophages from *in vitro* differentiation or from human resident M1 and M2 macrophages (Supplementary Fig. S6B; Supplementary Fig. S9B). Therefore, disrupting the CD47–SIRPα interaction with anti-CD47 likely promotes the ability of both M1 and M2 macrophages to clear cancer cells. These data indicate that ZL-1201 treatment can be especially beneficial in solid tumors that are highly enriched in suppressive TAM/M2 macrophages.

Notably, *in vivo* efficacy studies for ZL-1201 in combination with mAbs with or without chemotherapeutics were performed on BALB/c nude mice that lack functional T cells and have low NK-cell populations. Therefore, the observed combinational antitumor effects were mainly dependent on macrophages. Flow cytometry indicated that M2 is the main macrophage subtype within murine xenografts, and we observed that ZL-1201 can specifically convert M2 macrophages into phagocytic cells *in vitro*. Therefore, ZL-1201’s impact may be more significant in immune-competent environments, representative of human patients, because TAMs interact and cross-talk with other immune cells in the tumor microenvironment, including T cells, dendritic cells, and NK cells. Consistently, Tsao and colleagues identified that treatment with anti-HER2 and anti-CD47 enhanced macrophage polarization toward a proinflammatory and antitumor phenotype. However, hyperphagocytic macrophages adopted a regulatory genetic signature highlighted by expression of wound repair and extracellular matrix remodeling genes (16). As such, it remains to be seen how prolonged treatment with mAbs and anti-CD47 therapeutics will remodel the solid tumor environment, and whether alternative combination strategies will be needed.

In a tumor model of hematologic malignancy, ZL-1201 inhibited tumor growth in a dose-dependent manner, and 10 mg/kg of ZL-1201 achieved more than 80% TGI (Fig. 1F). In contrast, limited TGI (<50%) was observed across solid tumor models treated with 10 mg/kg of ZL-1201 (Supplementary Fig. S7), suggesting that CD47 blockade may promote antitumor activities more efficiently in lymphoma than in solid tumors. Furthermore, anti-CD47 in combination with rituximab achieved maximal and durable antitumor activity in a preclinical setting (18). Anti-CD47 combined with azacytidine in acute myeloid leukemia achieved significant anti-tumor activities in preclinical and clinical settings (7, 8). In contrast, ZL-1201 in combination with either mAbs or chemotherapeutics only achieved certain levels of combinatorial antitumor effects in xenograft studies, while ZL-1201 in combination with both mAb and chemotherapy achieved the maximal antitumor effects in a variety of solid tumor models. Collectively, these results suggest that the composition of solid tumors and the associated tumor microenvironment may differ from that of hematologic malignancies, indicative of a more complex microenvironment and higher barrier to therapeutic efficacy. Therefore, a triple combination might be required to achieve maximal antitumor efficacy in solid tumors.

Currently, a phase I dose-escalation study is underway to assess the safety, tolerability, pharmacodynamics, and pharmacokinetics of ZL-1201 in patients with lymphoma and solid tumors (NCT04257617).

## Supplementary Material

Figure S1 ZL-1201 binding to CD47

Figure S2 CD47 expression and phagocytosis by ZL-1201

Figure S3 In vitro phagocytosis by ZL-1201

Figure S4 Fc-dependent synergy of ZL-1201

Figure S5 Macrophage content in TCGA

Figure S6 Differentiation of macrophages

Figure S7 Individual tumor volumes

Figure S8 Body weights of mice in HCC1954(A), SKOV3(B), ST-02-0077(C), FaDu (D), and Raji(E) xenograft efficacy studies shown in Figure 5 at days post-treatment as shown

Figure S9 M1/M2 modulation by ZL-1201
