# Supplementary material for: CD47-blocking Antibody ZL-1201 Promotes Tumor-associated Macrophage Phagocytic Activity and Enhances the Efficacy of the Therapeutic Antibodies and Chemotherapy
Source: Cancer Res Commun. 2022 Nov 10;2(11):1404–17. doi: 10.1158/2767-9764.CRC-22-0266 (PMC10035405; doi:10.1158/2767-9764.CRC-22-0266)
Supplement: Figure S1 — ZL-1201 binding to CD47 [file crc-22-0266-s01.pdf]

Figure S1

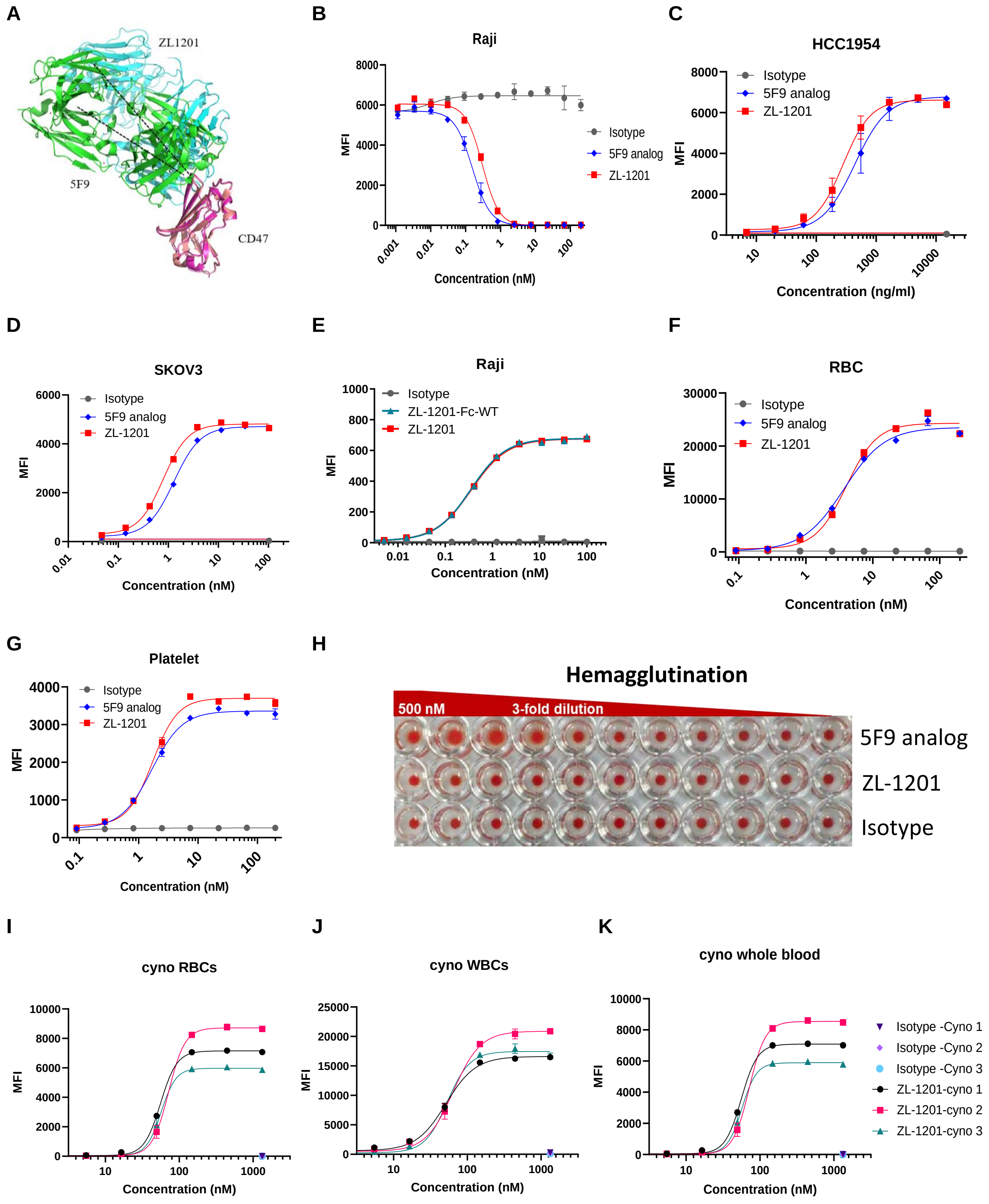
A
ZL1201
5F9
CD47
B
Raji
MFI
Concentration (nM)
Isotype
5F9 analog
ZL-1201
C
HCC1954
MFI
Concentration (ng/ml)
Isotype
5F9 analog
ZL-1201
D
SKOV3
MFI
Concentration (nM)
Isotype
5F9 analog
ZL-1201
E
Raji
MFI
Concentration (nM)
Isotype
ZL-1201-Fc-WT
ZL-1201
F
RBC
MFI
Concentration (nM)
Isotype
5F9 analog
ZL-1201
G
Platelet
MFI
Concentration (nM)
Isotype
5F9 analog
ZL-1201
H
Hemagglutination
500 nM
3-fold dilution
5F9 analog
ZL-1201
Isotype
I
cyno RBCs
MFI
Concentration (nM)
J
cyno WBCs
MFI
Concentration (nM)
K
cyno whole blood
MFI
Concentration (nM)
Isotype -Cyno 1
Isotype -Cyno 2
Isotype -Cyno 3
ZL-1201-cyno 1
ZL-1201-cyno 2
ZL-1201-cyno 3

Figure S1. A. Alignment of the complex structure of ZL-1201/CD47 and 5F9/CD47 complex (PDB 5IWL). Green: 5F9 diabody, cyan: ZL-1201 Fab. B. ZL-1201 and 5F9 prevents SIRPα binding to Raji cells in a dose-dependent manner. C and D. In vitro binding of isotype control, benchmark, and ZL-1201 with HCC1954 (C), and Raji (D) cells in a dose-dependent manner. E. In vitro binding of isotype control, ZL-1201-Fc-WT, and ZL-1201 with Raji cells in a dose-dependent manner. F and G. In vitro binding of isotype control, benchmark, and ZL-1201 with human red blood cells (RBC) (F) and platelets (G) in a dose-dependent manner. H. Hemagglutination assay of benchmark and ZL-1201 in dose dependent manner. Data are representative of two independent experiments. I-K. Binding of ZL-1201 to cynomolgus monkey RBCs (I), WBCs (J), and whole blood cells (K) (n = 3) in a dose-dependent manner. HuIgG4 isotype was used as a negative control at 1.34uM.
