# Supplementary material for: CD47-blocking Antibody ZL-1201 Promotes Tumor-associated Macrophage Phagocytic Activity and Enhances the Efficacy of the Therapeutic Antibodies and Chemotherapy
Source: Cancer Res Commun. 2022 Nov 10;2(11):1404–17. doi: 10.1158/2767-9764.CRC-22-0266 (PMC10035405; doi:10.1158/2767-9764.CRC-22-0266)
Supplement: Figure S2 — CD47 expression and phagocytosis by ZL-1201 [file crc-22-0266-s02.pdf]

Figure S2

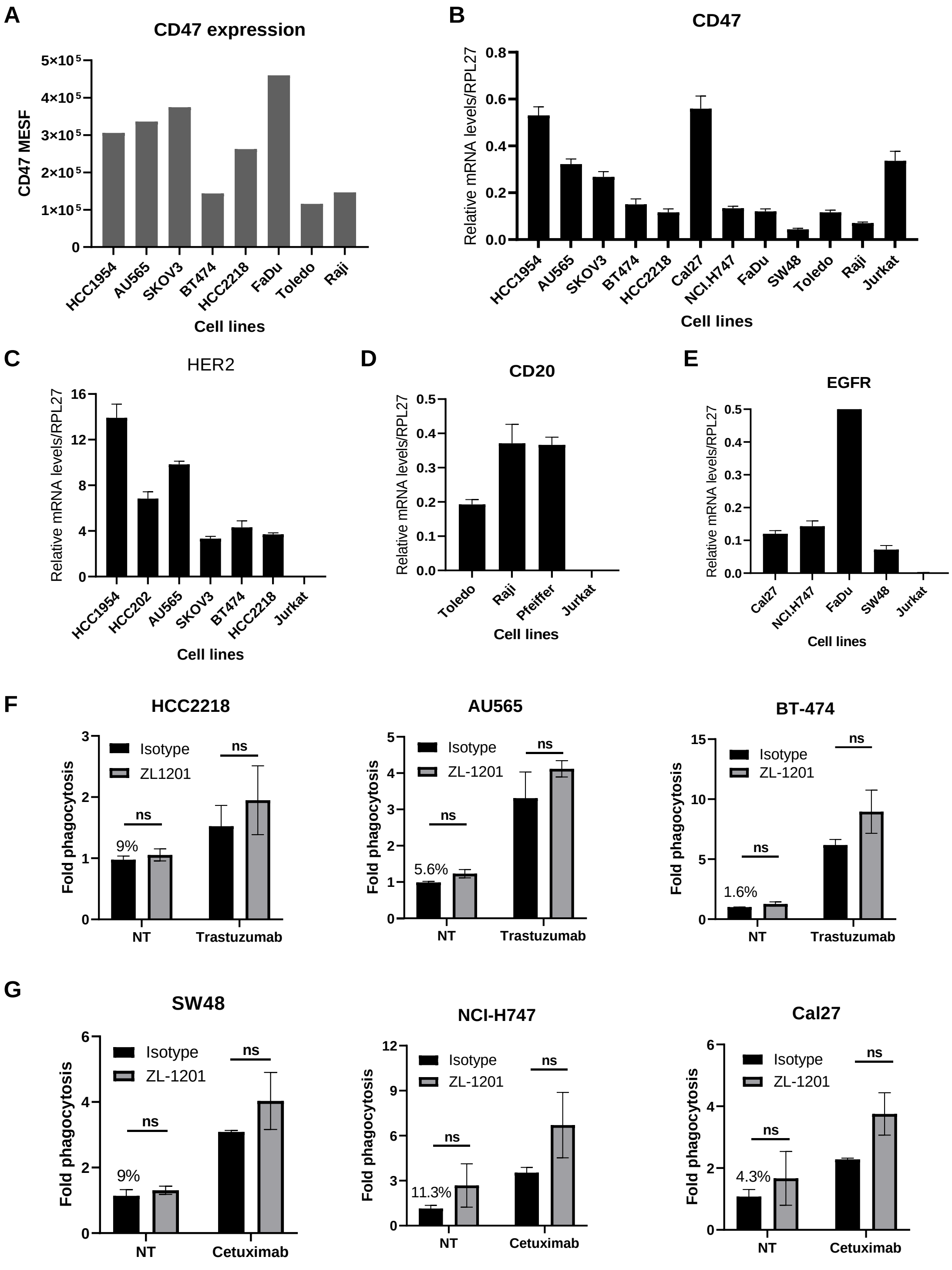
A
CD47 expression
CD47 MESF
Cell lines
HCC1954
AU565
SKOV3
BT474
HCC2218
FaDu
Toledo
Raji
B
CD47
Relative mRNA levels/RPL27
Cell lines
Cal27
NCI.H747
SW48
Jurkat
C
HER2
HCC202
D
CD20
Pfeiffer
E
EGFR
F
HCC2218
AU565
BT-474
Fold phagocytosis
Isotype
ZL1201
ZL-1201
ns
NT
Trastuzumab
9%
5.6%
1.6%
G
SW48
NCI-H747
Cal27
Cetuximab
11.3%
4.3%

Figure S2. A. CD47 surface expression levels in various cancer models measured by flow cytometry analysis. B-E, CD47 (B), HER2 (C), CD20 (D) and EGFR (E) transcript levels in cancer models were measured by RT-qPCR analysis relative to internal control RPL27. F, In vitro phagocytosis of HER2-amplified solid tumor cells including HCC2218, AU565, and BT-474 treated with isotype control (10μg/mL), ZL-1201 (10ug/mL), trastuzumab (0.1μg/mL), or their combination. G. In vitro phagocytosis of EGFR-driven SW48, NCI-H747, and Cal27 cells treated with isotype control (10μg/mL), ZL-1201 (10μg/mL), cetuximab (0.1μg/mL), or their combination. Fold phagocytosis in F and G was calculated relative to isotype in non-treated (NT) conditions, the raw phagocytosis percentages are indicated in each figure. Data was shown as mean ± SD; *$p<0.05$, **$p<0.01$, ***$p<0.001$.
