# Supplementary material for: CD47-blocking Antibody ZL-1201 Promotes Tumor-associated Macrophage Phagocytic Activity and Enhances the Efficacy of the Therapeutic Antibodies and Chemotherapy
Source: Cancer Res Commun. 2022 Nov 10;2(11):1404–17. doi: 10.1158/2767-9764.CRC-22-0266 (PMC10035405; doi:10.1158/2767-9764.CRC-22-0266)
Supplement: Figure S3 — In vitro phagocytosis by ZL-1201 [file crc-22-0266-s03.pdf]

Figure S3

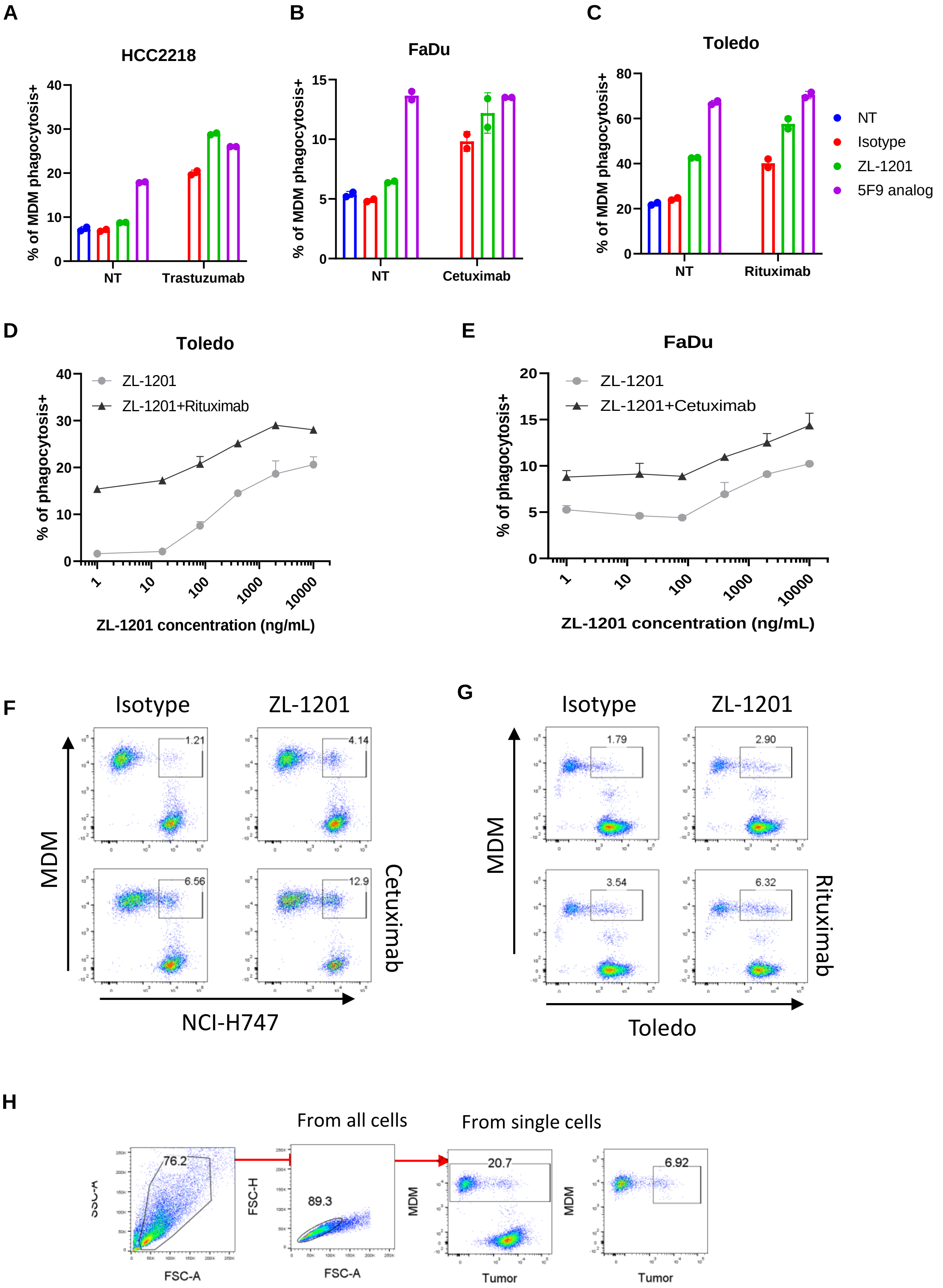
A
HCC2218
% of MDM phagocytosis+
NT
Trastuzumab
B
FaDu
% of MDM phagocytosis+
NT
Cetuximab
C
Toledo
% of MDM phagocytosis+
NT
Rituximab
NT
Isotype
ZL-1201
5F9 analog
D
Toledo
% of phagocytosis+
ZL-1201
ZL-1201+Rituximab
ZL-1201 concentration (ng/mL)
E
FaDu
% of phagocytosis+
ZL-1201
ZL-1201+Cetuximab
ZL-1201 concentration (ng/mL)
F
Isotype
ZL-1201
MDM
Cetuximab
NCI-H747
1.21
4.14
6.56
12.9
G
Isotype
ZL-1201
MDM
Rituximab
Toledo
1.79
2.90
3.54
6.32
H
From all cells
From single cells
SSC-A
FSC-A
76.2
FSC-H
FSC-A
89.3
MDM
Tumor
20.7
MDM
Tumor
6.92

Figure S3. A-C. In vitro phagocytosis of HCC2218 (A), FaDu (B), or Toledo (C) cells treated with isotype control (10µg/mL), ZL-1201 or benchmark (10ug/mL), trastuzumab, cetuximab, or rituximab (0.1µg/mL), or their combination. Data was shown as mean ± SD. D, E. In vitro phagocytosis of tumor cells Toledo (D) and FaDu (E) treated with increasing doses of ZL-1201 in the presence of 0.1µg/mL rituximab (D) or cetuximab (E). Data was shown as mean ± SD. F, G. Flow cytometry dot plots showing in vitro phagocytosis of ZL-1201 in combination with cetuximab on NCI-H747 cells (F) and with rituximab on Toledo cells (G). H. Flow plots showing gating strategies to define percentage of phagocytosis in the in vitro phagocytosis assays using differentiated MDM (CellTrace Red-labelled) and cancer cells (CellTrace Oregon Green-labelled).
