# Supplementary material for: CD47-blocking Antibody ZL-1201 Promotes Tumor-associated Macrophage Phagocytic Activity and Enhances the Efficacy of the Therapeutic Antibodies and Chemotherapy
Source: Cancer Res Commun. 2022 Nov 10;2(11):1404–17. doi: 10.1158/2767-9764.CRC-22-0266 (PMC10035405; doi:10.1158/2767-9764.CRC-22-0266)
Supplement: Figure S4 — Fc-dependent synergy of ZL-1201 [file crc-22-0266-s04.pdf]

Figure S4

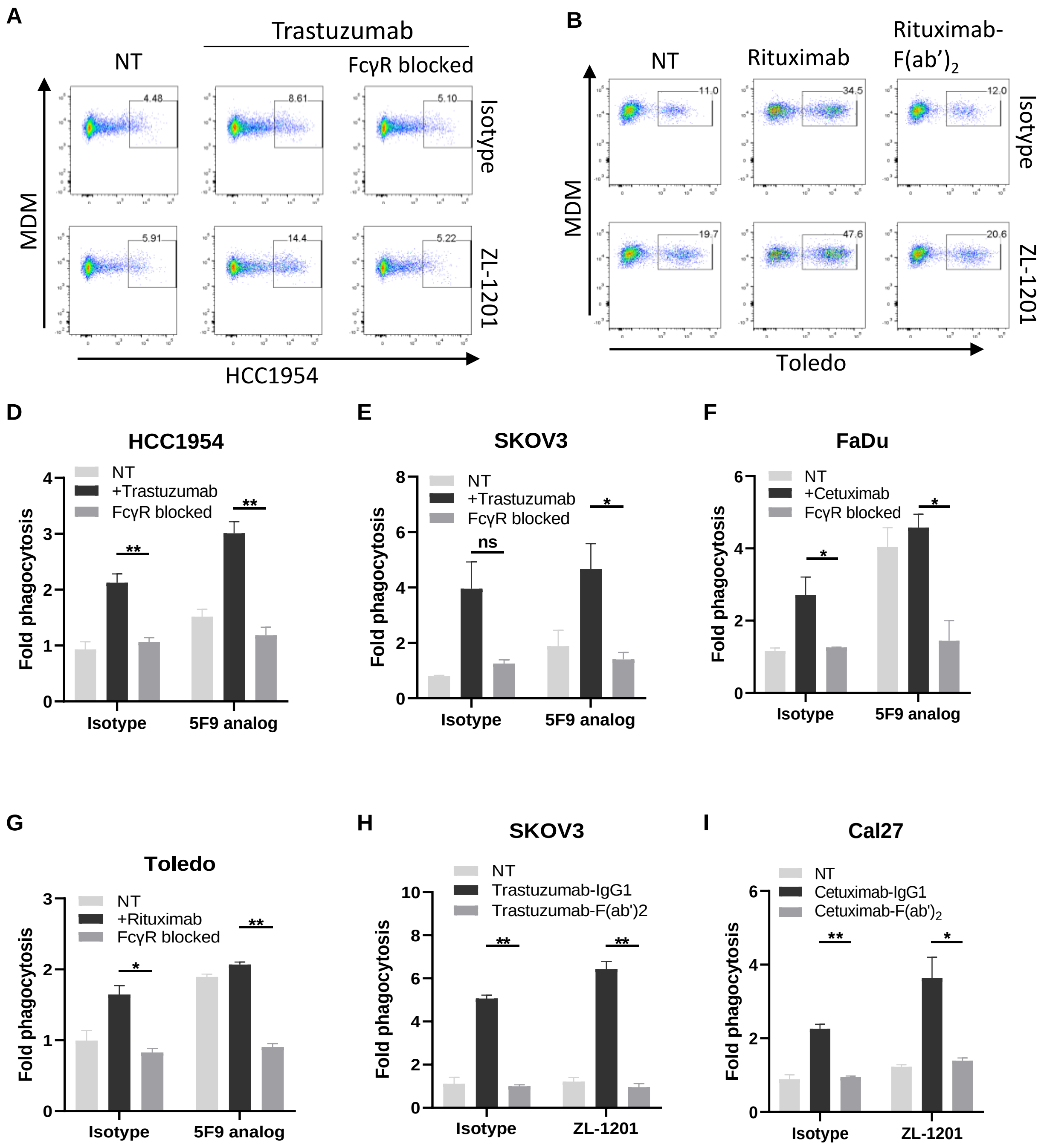
A
Trastuzumab
NT
FcγR blocked
4.48
8.61
5.10
5.91
14.4
5.22
Isotype
ZL-1201
MDM
HCC1954
B
NT
Rituximab
Rituximab-F(ab')2
11.0
34.5
12.0
19.7
47.6
20.6
Isotype
ZL-1201
MDM
Toledo
D
HCC1954
NT
+Trastuzumab
FcγR blocked
Fold phagocytosis
Isotype
5F9 analog
E
SKOV3
NT
+Trastuzumab
FcγR blocked
ns
Fold phagocytosis
Isotype
5F9 analog
F
FaDu
NT
+Cetuximab
FcγR blocked
Fold phagocytosis
Isotype
5F9 analog
G
Toledo
NT
+Rituximab
FcγR blocked
Fold phagocytosis
Isotype
5F9 analog
H
SKOV3
NT
Trastuzumab-IgG1
Trastuzumab-F(ab')2
Fold phagocytosis
Isotype
ZL-1201
I
Cal27
NT
Cetuximab-IgG1
Cetuximab-F(ab')2
Fold phagocytosis
Isotype
ZL-1201

Figure S4. A, Flow cytometry dot plots showing in vitro phagocytosis of ZL-1201 in combination with trastuzumab with or without FcγR blockade on HCC1954 cells. B. Flow cytometry dot plots showing in vitro phagocytosis of ZL-1201 in combination with rituximab-IgG1 or rituximab –(Fab')$_2$ on Toledo cells. D-G. In vitro phagocytosis of HCC1954 (D), SKOV3 (E), FaDu (F), or Toledo (G) treated with isotype or benchmark in combination with trastuzumab (D and E), cetuximab (F) or rituximab (G) (0.1μg/mL) with or without FcγR blockade. H, I. In vitro phagocytosis of SKOV3 (H) and Cal27 (I) treated with isotype control (NT), ZL-1201 in combination with trastuzumab-IgG1 or trastuzumab –F(ab')$_2$ (H); or cetuximab-IgG1 or cetuximab–F(ab')$_2$ (I). Data shown as mean ± SD, *$p<0.05$, **$p<0.01$.
