# Supplementary material for: CD47-blocking Antibody ZL-1201 Promotes Tumor-associated Macrophage Phagocytic Activity and Enhances the Efficacy of the Therapeutic Antibodies and Chemotherapy
Source: Cancer Res Commun. 2022 Nov 10;2(11):1404–17. doi: 10.1158/2767-9764.CRC-22-0266 (PMC10035405; doi:10.1158/2767-9764.CRC-22-0266)
Supplement: Figure S5 — Macrophage content in TCGA [file crc-22-0266-s05.pdf]

Figure S5

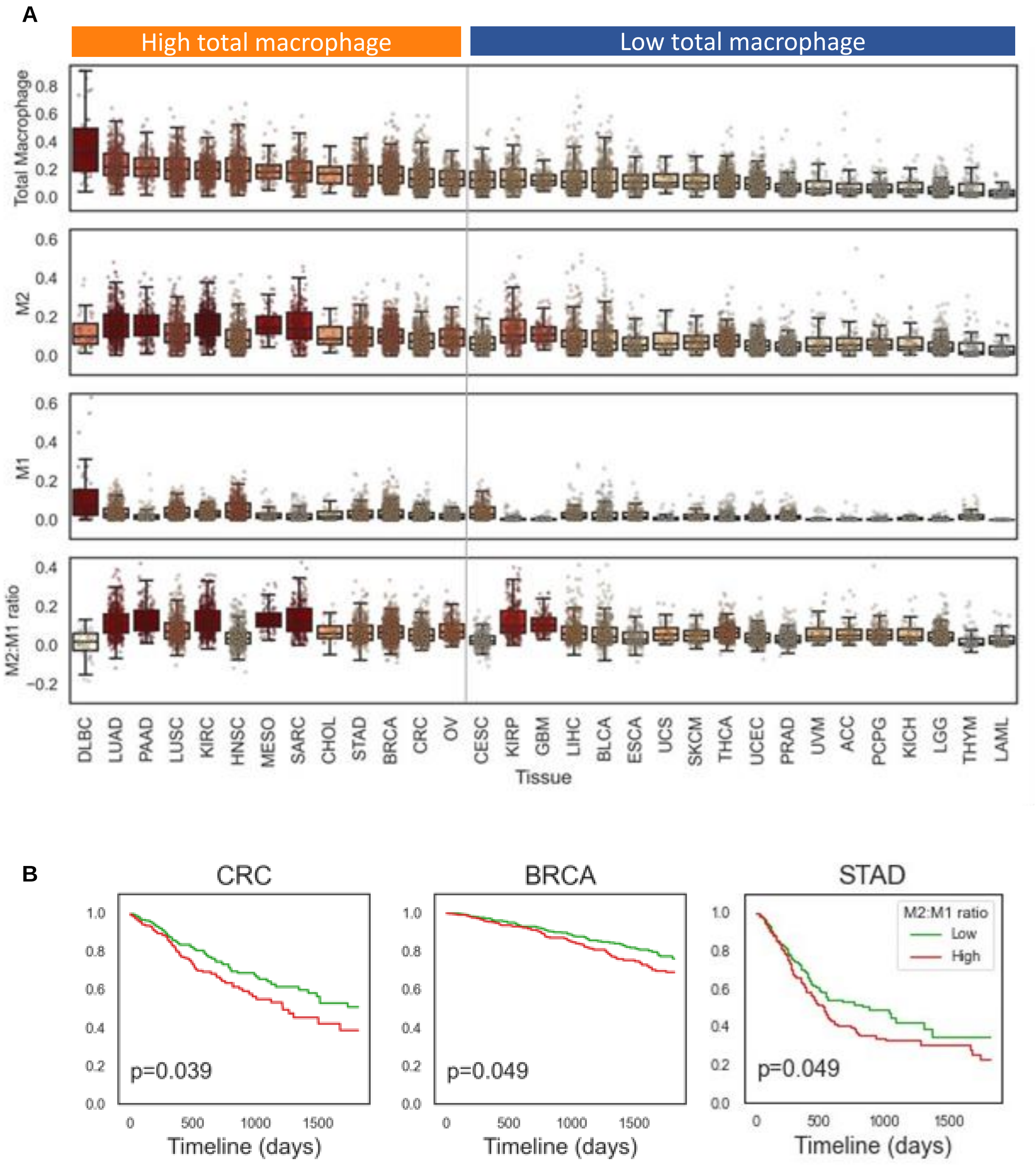
A
High total macrophage
Low total macrophage
Total Macrophage
M2
M1
M2:M1 ratio
DLBC LUAD PAAD LUSC KIRC HNSC MESO SARC CHOL STAD BRCA CRC OV CESC KIRP GBM LIHC BLCA ESCA UCS SKCM THCA UCEC PRAD UVM ACC PCPG KICH LGG THYM LAML
Tissue
B
CRC
BRCA
STAD
p=0.039
p=0.049
p=0.049
M2:M1 ratio
Low
High
Timeline (days)

Figure S5. A. Macrophage biomarker landscape across >11K TCGA patients. For each of four biomarkers (total macrophage score, M2, M1, and M2:M1 ratio), each patient is displayed as a point and each tissue type is summarized as a boxplot. Points and boxes are colored from white to red based on median value for the tissue type. The top row shows the total macrophage scores *(M0+M1+M2* from CIBERSORT-ABS), the second and third rows depict the M2 and M1 macrophage subsets, and the fourth row shows the ratio between M2 and M1 macrophages (as log2(M2+1) – log2(M1+1)). The vertical grey line across all rows shows which tissue types were considered macrophage-high (left) and low (right). B. Kaplan-Meier plots of M2:M1 ratio–high versus M2:M1 ratio–low subgroups in colorectal (CRC), breast (BRCA), and gastric (STAD) cancer types with poorer progression-free interval (PFI) across a 5-year duration.
