# Supplementary material for: CD47-blocking Antibody ZL-1201 Promotes Tumor-associated Macrophage Phagocytic Activity and Enhances the Efficacy of the Therapeutic Antibodies and Chemotherapy
Source: Cancer Res Commun. 2022 Nov 10;2(11):1404–17. doi: 10.1158/2767-9764.CRC-22-0266 (PMC10035405; doi:10.1158/2767-9764.CRC-22-0266)
Supplement: Figure S6 — Differentiation of macrophages [file crc-22-0266-s06.pdf]

Figure S6

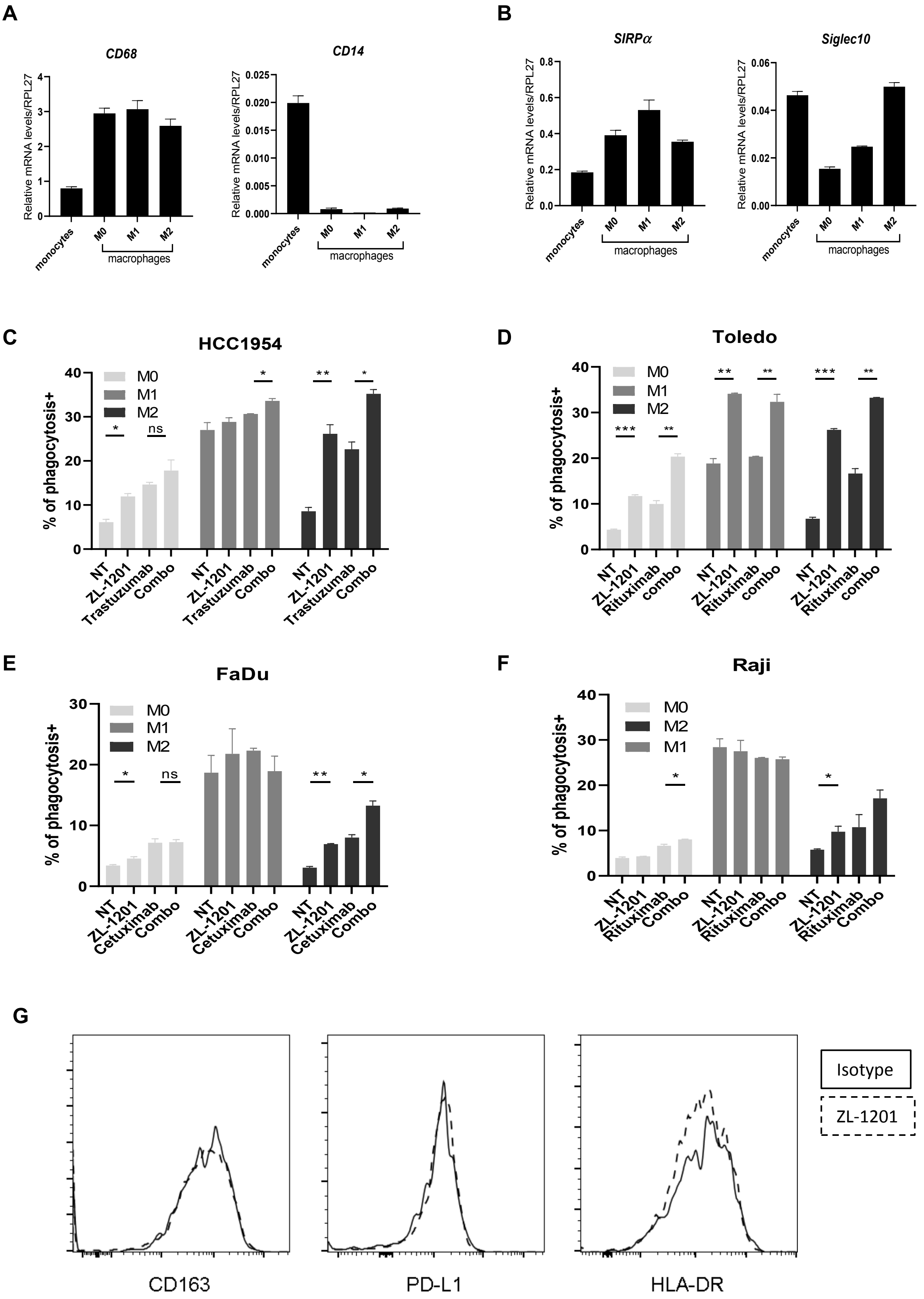
A
CD68
CD14
Relative mRNA levels/RPL27
monocytes
M0
M1
M2
macrophages
B
SIRPα
Siglec10
C
HCC1954
D
Toledo
E
FaDu
F
Raji
M0
M1
M2
% of phagocytosis+
NT
ZL-1201
Trastuzumab
Rituximab
Cetuximab
Combo
combo
ns
G
CD163
PD-L1
HLA-DR
Isotype
ZL-1201

Figure S6. A. CD68 and CD14 transcript levels in monocytes and differentiated M0, M1 and M2 macrophages. B. Siglec10 and SIRPα transcript levels in monocytes and differentiated M0, M1 and M2 macrophages. C-F. in vitro phagocytosis using HCC1954 (C), Toledo (D), FaDu (E), and Raji (F) cancer cells and differentiated M0, M1, or M2 macrophages under the treatment conditions as indicated. Percentage (%) phagocytosis was shown as mean ± SD. Student t-tests were performed comparing +/- ZL-1201, *p<0.05, **p<0.01, ***p<0.001. G. Flow histogram showing CD163, PD-L1, and HLA-DR levels in M2 macrophages treated with either isotype (solid line) or ZL-1201 (dotted line) after phagocytosis of FaDu cells.
