# Supplementary material for: CD47-blocking Antibody ZL-1201 Promotes Tumor-associated Macrophage Phagocytic Activity and Enhances the Efficacy of the Therapeutic Antibodies and Chemotherapy
Source: Cancer Res Commun. 2022 Nov 10;2(11):1404–17. doi: 10.1158/2767-9764.CRC-22-0266 (PMC10035405; doi:10.1158/2767-9764.CRC-22-0266)
Supplement: Figure S7 — Individual tumor volumes [file crc-22-0266-s07.pdf]

Figure S7

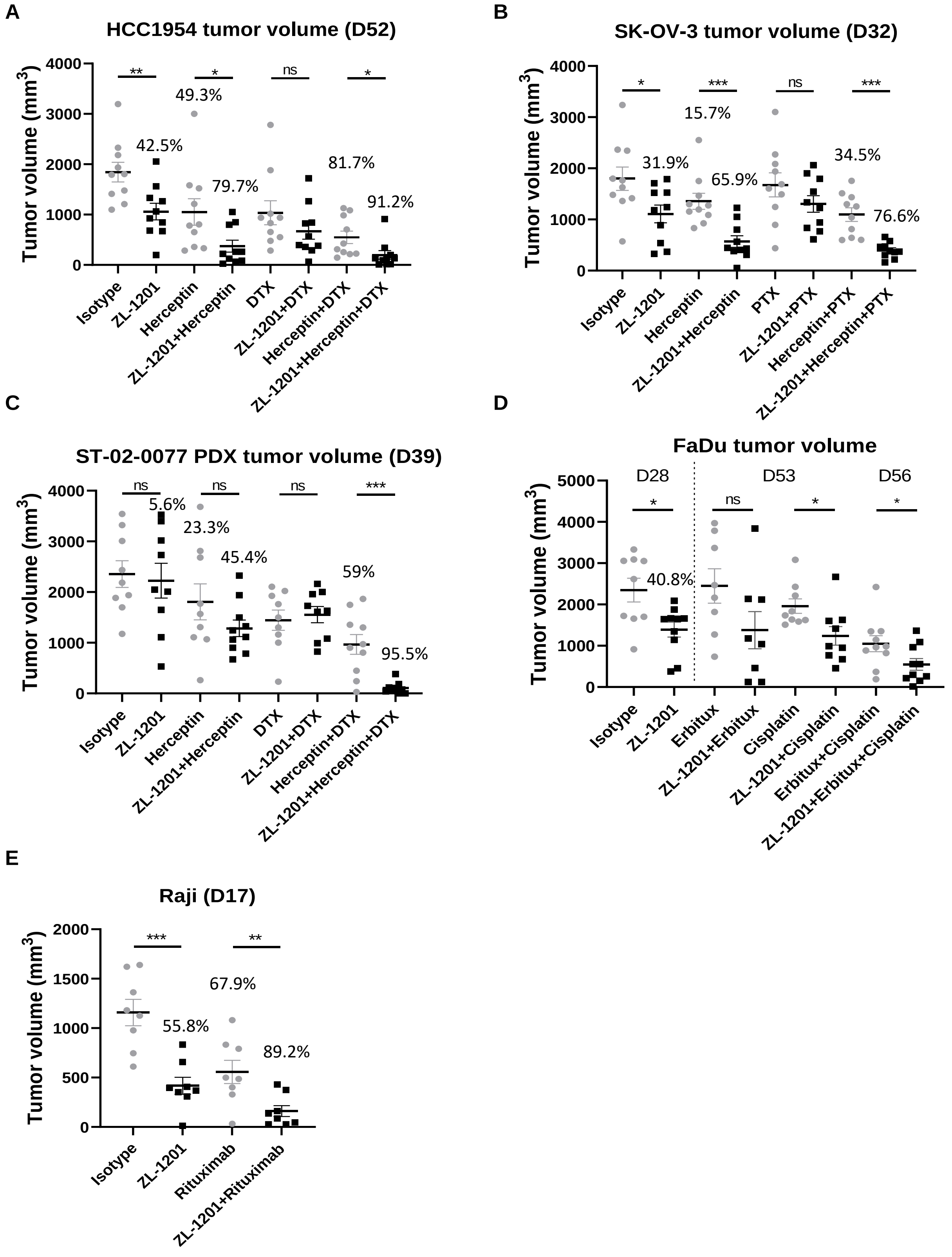
A
HCC1954 tumor volume (D52)
Tumor volume (mm3)
4000
3000
2000
1000
0
**
*
ns
*
42.5%
49.3%
79.7%
81.7%
91.2%
Isotype
ZL-1201
Herceptin
ZL-1201+Herceptin
DTX
ZL-1201+DTX
Herceptin+DTX
ZL-1201+Herceptin+DTX
B
SK-OV-3 tumor volume (D32)
Tumor volume (mm3)
4000
3000
2000
1000
0
*
***
ns
***
31.9%
15.7%
65.9%
34.5%
76.6%
Isotype
ZL-1201
Herceptin
ZL-1201+Herceptin
PTX
ZL-1201+PTX
Herceptin+PTX
ZL-1201+Herceptin+PTX
C
ST-02-0077 PDX tumor volume (D39)
Tumor volume (mm3)
4000
3000
2000
1000
0
ns
ns
ns
***
5.6%
23.3%
45.4%
59%
95.5%
Isotype
ZL-1201
Herceptin
ZL-1201+Herceptin
DTX
ZL-1201+DTX
Herceptin+DTX
ZL-1201+Herceptin+DTX
D
FaDu tumor volume
D28
D53
D56
Tumor volume (mm3)
5000
4000
3000
2000
1000
0
*
ns
*
*
40.8%
Isotype
ZL-1201
Erbitux
ZL-1201+Erbitux
Cisplatin
ZL-1201+Cisplatin
Erbitux+Cisplatin
ZL-1201+Erbitux+Cisplatin
E
Raji (D17)
Tumor volume (mm3)
2000
1500
1000
500
0
***
**
67.9%
55.8%
89.2%
Isotype
ZL-1201
Rituximab
ZL-1201+Rituximab

Figure S7. A. Individual tumor volumes of HCC1954 breast cancer xenograft in efficacy study shown in Figure 6A at day 52 post-treatment as shown. B. Individual tumor volumes of SKOV3 ovarian cancer xenograft in efficacy study shown in Figure 6B at day 32 post-treatment as shown. C. Individual tumor volumes of ST-02-0077 gastric PDX model in efficacy study shown in Figure 6C at day 39 post-treatment as shown. D. Individual tumor volumes of FaDu HNSCC xenograft in efficacy study shown in Figure 6D at day 28, 53 or 56 post-treatments as shown. E. Individual tumor volumes of Raji lymphoma xenograft in efficacy study shown in Figure 6E at day 17 post-treatment as shown. Percentage in the figure show tumor growth inhibition (%) relative to isotype treatment. TGI in D was calculated on D28 post-treatment. *$p<0.05$, **$p<0.01$, ***$p<0.001$.
