# Supplementary material for: CD47-blocking Antibody ZL-1201 Promotes Tumor-associated Macrophage Phagocytic Activity and Enhances the Efficacy of the Therapeutic Antibodies and Chemotherapy
Source: Cancer Res Commun. 2022 Nov 10;2(11):1404–17. doi: 10.1158/2767-9764.CRC-22-0266 (PMC10035405; doi:10.1158/2767-9764.CRC-22-0266)
Supplement: Figure S8 — Body weights of mice in HCC1954(A), SKOV3(B), ST-02-0077(C), FaDu (D), and Raji(E) xenograft efficacy studies shown in Figure 5 at days post-treatment as shown [file crc-22-0266-s08.pdf]

Figure S8

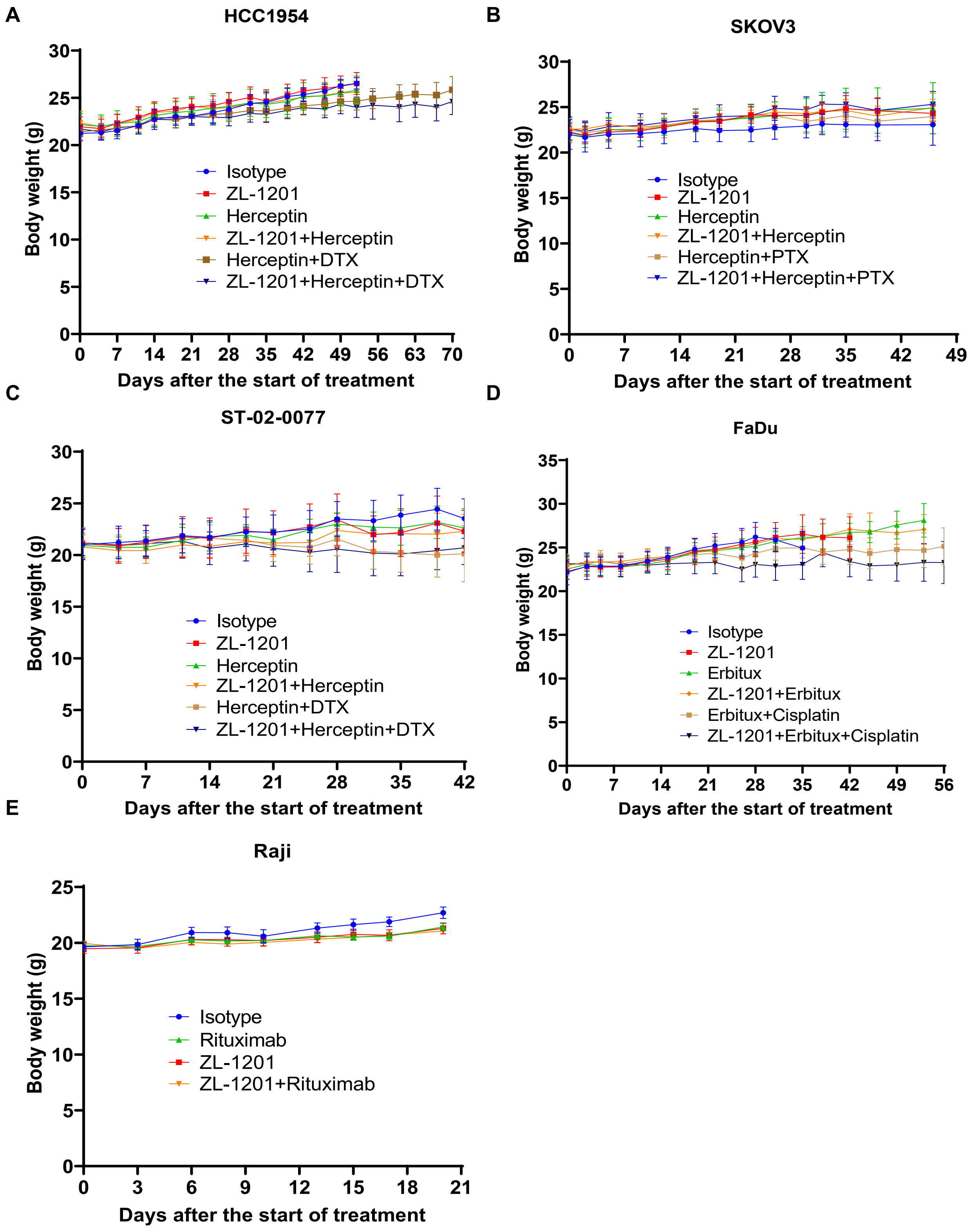
A
HCC1954
Body weight (g)
Days after the start of treatment
Isotype
ZL-1201
Herceptin
ZL-1201+Herceptin
Herceptin+DTX
ZL-1201+Herceptin+DTX
B
SKOV3
Body weight (g)
Days after the start of treatment
Isotype
ZL-1201
Herceptin
ZL-1201+Herceptin
Herceptin+PTX
ZL-1201+Herceptin+PTX
C
ST-02-0077
Body weight (g)
Days after the start of treatment
Isotype
ZL-1201
Herceptin
ZL-1201+Herceptin
Herceptin+DTX
ZL-1201+Herceptin+DTX
D
FaDu
Body weight (g)
Days after the start of treatment
Isotype
ZL-1201
Erbitux
ZL-1201+Erbitux
Erbitux+Cisplatin
ZL-1201+Erbitux+Cisplatin
E
Raji
Body weight (g)
Days after the start of treatment
Isotype
Rituximab
ZL-1201
ZL-1201+Rituximab

Figure S8. A-E. Body weights of mice in HCC1954(A), SKOV3(B), ST-02-0077(C), FaDu (D), and Raji(E) xenograft efficacy studies shown in Figure 5 at days post-treatment as shown. Data are shown as mean ± SEM.
