# Supplementary material for: CD47-blocking Antibody ZL-1201 Promotes Tumor-associated Macrophage Phagocytic Activity and Enhances the Efficacy of the Therapeutic Antibodies and Chemotherapy
Source: Cancer Res Commun. 2022 Nov 10;2(11):1404–17. doi: 10.1158/2767-9764.CRC-22-0266 (PMC10035405; doi:10.1158/2767-9764.CRC-22-0266)
Supplement: Figure S9 — M1/M2 modulation by ZL-1201 [file crc-22-0266-s09.pdf]

Figure S9

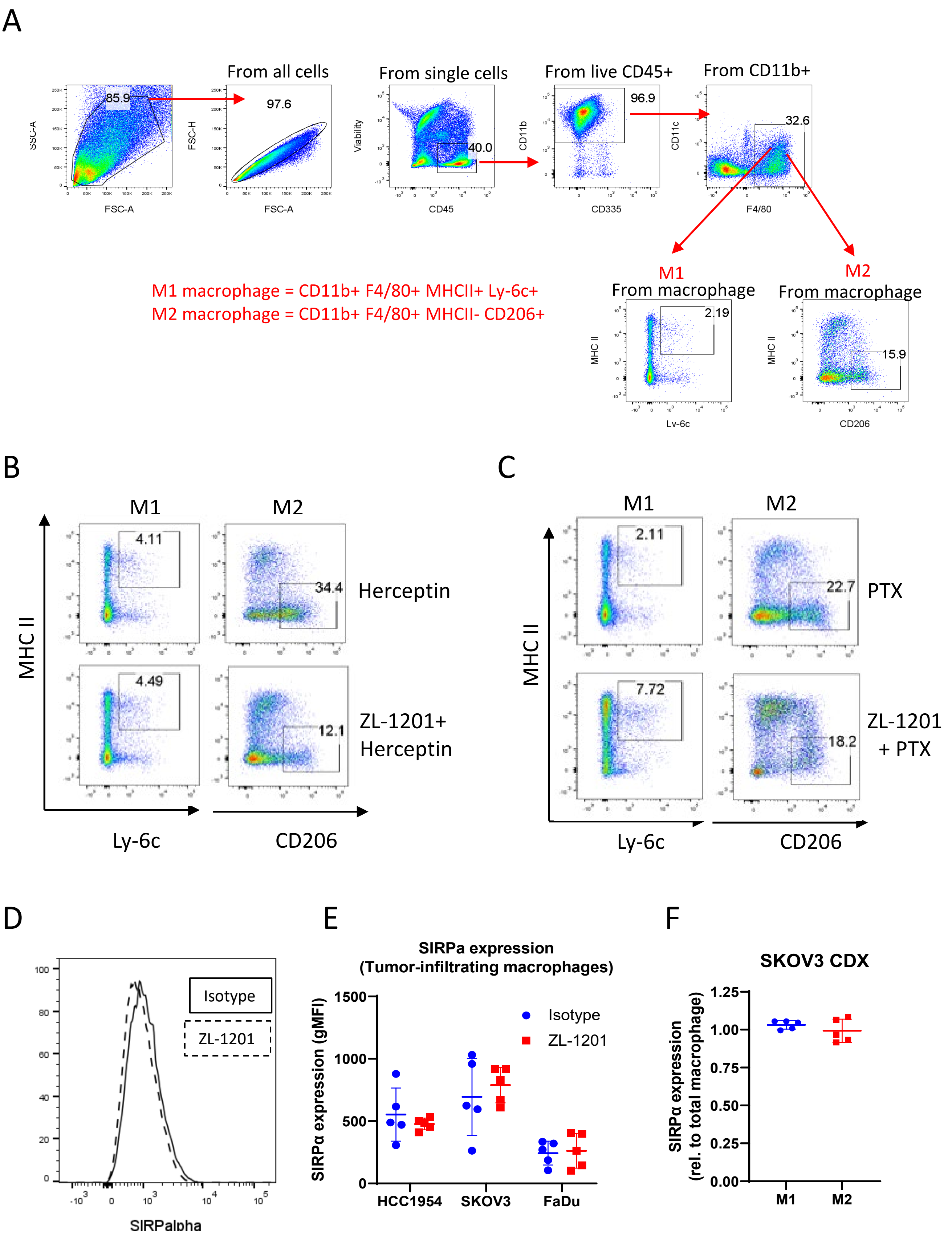

A
From all cells
From single cells
From live CD45+
From CD11b+
85.9
97.6
40.0
96.9
32.6
FSC-A
FSC-H
Viability
CD45
CD11b
CD335
CD11c
F4/80
M1
From macrophage
M2
From macrophage
2.19
15.9
MHC II
Ly-6c
CD206
M1 macrophage = CD11b+ F4/80+ MHCII+ Ly-6c+
M2 macrophage = CD11b+ F4/80+ MHCII- CD206+
B
M1
M2
4.11
34.4
Herceptin
4.49
12.1
ZL-1201+
Herceptin
MHC II
Ly-6c
CD206
C
M1
M2
2.11
22.7
PTX
7.72
18.2
ZL-1201
+ PTX
MHC II
Ly-6c
CD206
D
Isotype
ZL-1201
SIRPalpha
E
SIRPa expression
(Tumor-infiltrating macrophages)
SIRPα expression (gMFI)
Isotype
ZL-1201
HCC1954
SKOV3
FaDu
F
SKOV3 CDX
SIRPα expression
(rel. to total macrophage)
M1
M2

Figure S9. A. Flow plots showing gating strategies and markers used to define immune subtypes including M1 and M2 macrophages. B. Flow plots showing M1 and M2 macrophages in one out of five SKOV3 xenograft tumor tissues treated with Herceptin and ZL-1201 + Herceptin. C. Flow plots showing M1 and M2 macrophages in one out of five SKOV3 xenograft tumor tissues treated with paclitaxel (PTX) and ZL-1201 + PTX. D. Flow histogram showing SIRPa levels in SKOV3 xenograft tumor tissues treated with either isotype or ZL-1201. E. SIRPa levels on tumor-infiltrating macrophages analyzed by FACs analysis in HCC1954, SKOV3, and FaDu xenograft tumor tissues (n = 5) treated with either isotype or ZL-1201. E. SIRPa levels in M1 and M2 macrophages from SKOV3 xenograft tumor tissues (n = 5) relative to total macrophages. For E and F, data was shown as mean ± SEM.
